# A variant-proof SARS-CoV-2 vaccine targeting HR1 domain in S2 subunit of spike protein

**DOI:** 10.1038/s41422-022-00746-3

**Published:** 2022-11-10

**Authors:** Wei Pang, Ying Lu, Yan-Bo Zhao, Fan Shen, Chang-Fa Fan, Qian Wang, Wen-Qiang He, Xiao-Yan He, Ze-Kai Li, Tao-Tao Chen, Cui-Xian Yang, You-Zhi Li, Si-Xuan Xiao, Zu-Jiang Zhao, Xu-Sheng Huang, Rong-Hua Luo, Liu-Meng Yang, Mi Zhang, Xing-Qi Dong, Ming-Hua Li, Xiao-Li Feng, Qing-Cui Zhou, Wang Qu, Shibo Jiang, Songying Ouyang, Yong-Tang Zheng

**Affiliations:** 1 Key Laboratory of Animal Models and Human Disease Mechanisms of the Chinese Academy of Sciences/Key Laboratory of Bioactive Peptides of Yunnan Province, KIZ-CUHK Joint Laboratory of Bioresources and Molecular Research in Common Diseases, Kunming Institute of Zoology, Chinese Academy of Sciences, Kunming, Yunnan China; 2 University of the Chinese Academy of Sciences, Beijing, China; 3 The Key Laboratory of Innate Immune Biology of Fujian Province, Provincial University Key Laboratory of Cellular Stress Response and Metabolic Regulation, Biomedical Research Center of South China, Key Laboratory of OptoElectronic Science and Technology for Medicine of Ministry of Education, College of Life Sciences, Fujian Normal University, Fuzhou, Fujian China; 4 Division of Animal Model Research, Institute for Laboratory Animal Resources, National Institutes for Food and Drug Control, Beijing, China; 5 Key Laboratory of Medical Molecular Virology (MOE/NHC/CAMS), Shanghai Institute of Infectious Disease and Biosecurity, School of Basic Medical Sciences, Fudan University, Shanghai, China; 6 Yunnan Provincial Infectious Disease Hospital, Kunming, Yunnan China; 7 Kunming National High-level Biosafety Research Center for Non-human Primates, Center for Biosafety Mega-Science, Kunming Institute of Zoology, Chinese Academy of Sciences, Kunming, Yunnan China

**Keywords:** Mechanisms of disease, Molecular modelling

## Abstract

The emerging SARS-CoV-2 variants, commonly with many mutations in S1 subunit of spike (S) protein are weakening the efficacy of the current vaccines and antibody therapeutics. This calls for the variant-proof SARS-CoV-2 vaccines targeting the more conserved regions in S protein. Here, we designed a recombinant subunit vaccine, HR121, targeting the conserved HR1 domain in S2 subunit of S protein. HR121 consisting of HR1–linker1–HR2–linker2–HR1, is conformationally and functionally analogous to the HR1 domain present in the fusion intermediate conformation of S2 subunit. Immunization with HR121 in rabbits and rhesus macaques elicited highly potent cross-neutralizing antibodies against SARS-CoV-2 and its variants, particularly Omicron sublineages. Vaccination with HR121 achieved near-full protections against prototype SARS-CoV-2 infection in hACE2 transgenic mice, Syrian golden hamsters and rhesus macaques, and effective protection against Omicron BA.2 infection in Syrian golden hamsters. This study demonstrates that HR121 is a promising candidate of variant-proof SARS-CoV-2 vaccine with a novel conserved target in the S2 subunit for application against current and future SARS-CoV-2 variants.

## Introduction

SARS-CoV-2 and its emerging variants of concern (VOCs), particularly Omicron sublineages with strong immune evasion and transmission, pose substantial challenges to the control of COVID-19 pandemic,^1–3^ thus calling for the development of variant-proof SARS-CoV-2 or pan-sarbecovirus vaccines.^4,5^

The step of SARS-CoV-2 entry into host cells is a main target for the development of vaccines and therapeutic approaches. The spike (S) protein mediates SARS-CoV-2 entry by binding its S1 subunit to the host receptor angiotensin-converting enzyme 2 (ACE2), and subsequently promoting viral and cellular membrane fusion by its S2 subunit, leading to the release of viral genome into the cytoplasm. The S1 subunit, particularly its receptor-binding domain (RBD) and N-terminal domain (NTD), induces dominant neutralizing antibody (nAb) production in the host and serves as a prime antigen for vaccine design.^6–13^ However, selective pressure from the host acts on the S1 subunit in a manner that has increased the number of mutations in an equally growing number of new variants. This “domino” effect is steadily weakening the efficiency of some current antibodies and vaccines,^14–21^ and leading to constantly and possibly breakthrough infections in vaccinated individuals from some variants, such as Delta,^22^ Omicron BA.1 and its sublineages, including BA.2, BA.3, BA.4, and BA.5, as well as other emerging recombinant or hybrid variants.^3,23–26^

The S2 subunit, unlike S1 subunit, is buried inside the S protein in a prefusion conformation,^27,28^ and induces few nAbs against SARS-CoV-2 after viral infections^29–31^ or post vaccinations with its full amino acid sequence.^7,32^ The S2 subunit contains two important domains, heptad repeat 1 (HR1) and heptad repeat 2 (HR2), which are highly conserved among coronaviruses.^9,33,34^ According to the currently accepted membrane fusion model of class I enveloped viruses, such as HIV-1, influenza virus, and coronaviruses,^6,34–40^ fusion occurs when the hydrophobic fusion peptides (FPs) in the S2 homotrimer are inserted into the host cell membrane. Consequently, HR1 and HR2 adjacent to FP are instantly exposed to form a “fusion intermediate” or “prehairpin intermediate” conformation. Subsequently, three HR2 peptides move backward in an antiparallel manner and fold into the three surface grooves of the HR1 trimeric α-helical inner core, thus forming an irreversible six-α-helical bundle (6-HB) structure that maintains close contact between viral and cellular membranes and promotes membrane fusion. In this process, some HR2-derived peptides, binding to the HR1 trimer,^34,41–44^ and rare monoclonal antibodies (mAbs) isolated from COVID-19 convalescent individuals, with high affinities to HR2,^33^ can interfere with the conformational transition of the HR domains from fusion intermediate to post-fusion structure of 6-HB, thus demonstrating broad-spectrum antiviral activities against multiple SARS-CoV-2 variants, coronaviruses, and even HIV-1.^43^ Therefore, the conserved HR1 and HR2 domains, present in the fusion intermediate conformation of the S2 subunit, may serve as potential targets for vaccine development. However, previous reports on HIV-1^45^ and influenza viruses^46^ suggested that the fusion intermediate was transient and unstable, and its structure in enveloped viruses has not been resolved so far, making it difficult to design an immunogen capable of mimicking its conformation and evoking highly active nAbs in vivo. For instance, using core regions of HR1 and HR2, a lead study in HIV-1 had designed a 5-helix protein by linking three truncated HR1s and two truncated HR2s together (consisting of HR1–HR2–HR1–HR2–HR1). This protein lacked an HR2 helix; therefore, one surface groove in the HR1 trimeric inner core was unoccupied and could serve as HR1-based antigenic epitopes.^47^ However, the mAb targeting this vacancy only demonstrated weak anti-HIV-1 activities at subnanomolar ranges.^48^ We also showed that the 5-helix protein from human respiratory syncytial virus (hRSV) only elicited weak nAb titers to hRSV.^49^ Besides, some other studies on HIV-1 have developed some HR1-based trimers consisting of three different truncated HR1s, such as N35CCG-N13, (CCIZN36)3, (CCIPN36)3, and N46FdFc, but the nAbs induced by these proteins were proved similarly weak and unsatisfactory.^50–52^

In this study, we provided a new strategy to develop SARS-CoV-2 vaccines. We designed a recombinant protein HR121 from SARS-CoV-2, which could highly mimic the conformation of the HR1 trimeric inner core in the fusion intermediate of the S2 subunit. Immunization with HR121 induced potent broad-spectrum nAbs against SARS-CoV-2 and its main variants, including the current pandemic Omicron sublineages BA.1, BA.2, BA.3, BA.4/5. Furthermore, vaccination with this protein provided almost complete protection against the prototype SARS-CoV-2 (hereafter as SARS-CoV-2) infection in hACE2 transgenic mice, Syrian golden hamsters and rhesus macaques, and effective protection against Omicron BA.2 variant challenge in golden hamsters. Thus, this study demonstrates that the conserved HR1 domain in the S2 subunit, mimicking the fusion intermediate conformation, can serve as a new target for the development of variant-proof SARS-CoV-2 or pan-sarbecovirus vaccines.

## Results

### Design and characterization of the recombinant protein HR121

**Fig. 1:**
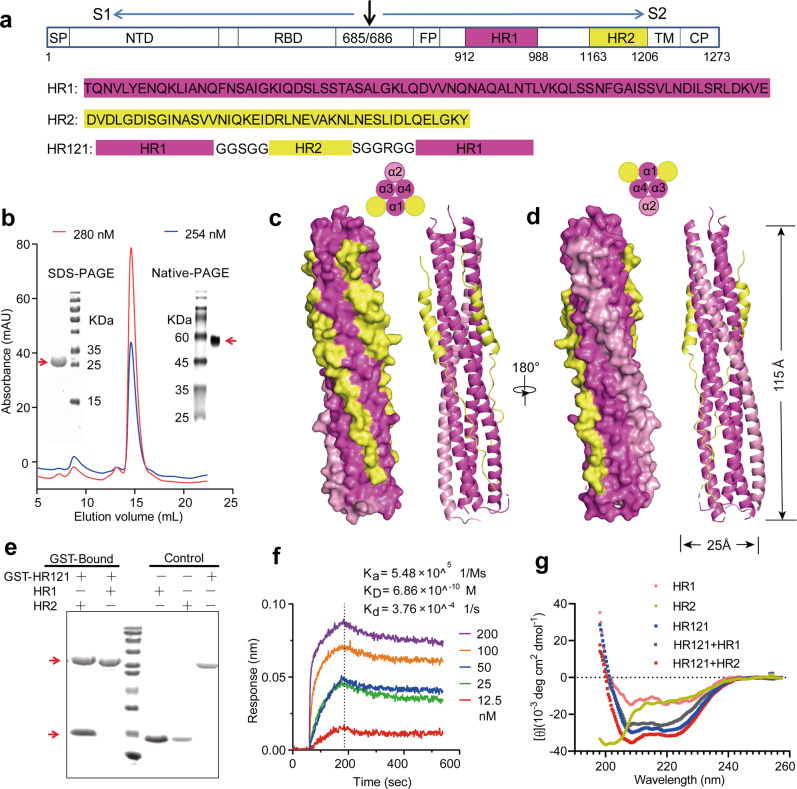
Design and characterization of the recombinant protein HR121. **a** Amino acid sequence of HR121. HR1 and HR2 peptide sequences were derived from the HR1 and HR2 domains, respectively, in the S protein of the prototype SARS-CoV-2 Wuhan-Hu-1 strain. HR121 monomer consists of HR1–linker 1–HR2–linker 2–HR1 segments (linker 1: GGSGG; linker 2: SGGRGG). Signal peptide (SP), N-terminal domain (NTD), and receptor-binding domain (RBD) in S1 subunit, and fusion peptide (FP), HR1 (aa 912–988), HR2 (aa 1163–1206), transmembrane domain (TM) and cytoplasmic domain (CP) in S2 subunit, were marked in the schematic diagram. **b** Purification of HR121. HR121 protein was purified using a Superdex 200 Increase 10/300 GL column; the profiles of the representative elution, SDS-PAGE and Native-PAGE were presented (the red arrows point the band of HR121 in SDS-PAGE and Native-PAGE gels); mAU in *y*-axis means milli-absorbance units. **c**, **d** Surface and cartoon representation of the atomic model of HR121 dimer. HR121 dimer consists of four HR1 (α1–α4) and two HR2. Front view (**c**) displayed the inner HR1 trimer (α1, α3 and α4) surrounded by two HR2s and one HR1 (α2), and back view (**d**) showed the two dominant-negative interfaces (between α2 and α3, α2 and α4) formed in the outer HR1 trimer (α2, α3 and α4). **e** GST pull-down assay showed that HR121 selectively bound to HR2, but not to HR1 (the red arrow at upper position points the band of GST-HR121, while red arrow at lower position points the band of HR2 or HR1). **f** Surface plasmon resonance recorded the profile of a real-time affinity of HR2 to HR121. **g** CD spectroscopic analysis showed that HR121 could interact with HR2 to form a more stable α-helix.

In our previous studies, using the truncated HR1 and HR2 sequences from HIV-1, hRSV, and SARS-CoV, we have separately designed three small recombinant HR121 proteins as fusion inhibitors against these viruses. These three proteins, with molecular weights of ~13 kDa, exhibited antiviral activities at nM–μM ranges, and showed interactions with HR2, implying that the motif of HR121 could mimic the function of HR1-based fusion intermediate.^53–55^ In this study, to improve the immunogenicity of HR121 and develop it as a novel antibody-based vaccine, we used the full amino acid sequences of HR domains derived from S2 subunit of SARS-CoV-2 Wuhan-Hu-1 strain, and linked HR1–HR2–HR1 together to produce a much larger recombinant protein, SARS-CoV-2-HR121 (abbreviated as HR121) (Fig. 1a). Initially, we attempted to design an immunogen of trimeric HR121, in which three HR1s and three HR2s form an HR1–HR2 trimer (6-HB scaffold), while three free HR1s would aggregate together, thereby mimicking the conformation of the HR1 trimeric inner core in the fusion intermediate, as predicted for the other three viruses using computer models.^53–55^

HR121 was abundantly expressed in *Escherichia coli* strain BL21 (DE3) in a soluble state, and was easily purified from lytic cellular supernatant by one step of elution on a Superdex 200 increase column (Fig. 1b). Every liter of the cultured cells could yield 10–20 mg of high-purity (> 95%) protein. The molecular weight of the HR121 monomer was determined to be 22 kDa by its amino acid sequence, which was confirmed by SDS-PAGE. However, it was unexpectedly observed to be a dimer in PBS by Native-PAGE (Fig. 1b). X-ray crystal diffraction in 2.41-Å resolution demonstrated that HR121 dimerized into a stable asymmetric 6-HB conformation with a rod-like shape (115 Å length and 25 Å diameter) (Fig. 1c, d). The HR121 dimer (or HR121) consists of a parallel HR1 tetrameric coiled-coil helix (the four HR1s were termed α1–α4), packed with two antiparallel HR2 helices (Supplementary information, Video S1, Fig. S1a, b). Each α-helix in HR121 shares a typical seven-amino acid wheel of 3–4 heptad repeats (the positions are denoted by letters “a” to “g”). The three internal HR1s (α1, α3, and α4) interact with each other via hydrophobic residues located at the “a” and “d” positions. Furthermore, they bind two exterior HR2s and one HR1 (α2) packed at their three hydrophobic surface grooves using residues located at the “e” and “g” positions, commonly found in the 6-helix coiled-coil proteins. Compared with the post-fusion structure of SARS-CoV-2 6-HB (PDB code: 6LXT),^34^ HR121 displays a very similar conformation and amino acid residue interaction, except that a parallel HR1 (α2) in HR121 replaces an antiparallel HR2 in 6-HB, via its residues located at the “a” and “e” positions interacting with residues located at the “e” and “g” positions in the other two HR1s (α4 and α3), respectively (Supplementary information, Figs. S1b, c, S2a–d). This results in a 0.5-coil dislocation in this HR1 (α2) helix, compared to the other three HR1s (α1, α3, and α4) (Supplementary information, Fig. S1d). As such, the four HR1 helices in HR121 combine to form two sets of trimeric HR1s. The inner HR1 trimer (α1, α3 and α4) are filled with two HR2s and one HR1 (α2) in its surface hydrophobic grooves (Fig. 1c). The outer HR1 trimer (α2, α3 and α4) also bind two HR2s and one HR1 (α1), but its two surface grooves formed by two adjacent HR1s (between α2 and α3, α2 and α4) remain unoccupied (Fig. 1d). These two exposed surface grooves show higher charged surfaces than the counterparts in the HR1 trimer of SARS-CoV-2 6-HB post-fusion structure, explaining the high solubility of HR121 (Supplementary information, Fig. S2e, f). Notably, these two vacancies may provide two possible binding sites for exogenous HR2s, and are, therefore, expected to create efficient antigenic epitopes to elicit nAbs targeting the HR1 trimeric core in the S2 fusion intermediate.

To verify the above conjecture, we first explored the affinity between HR121 and HR2 or HR1. Both HR1 and HR2 were expressed in *E. coli* BL21 containing pET-30a vector, thus each of them containing a redundant 52 amino acid sequence from the vector. Using a GST pull-down assay, we observed that HR121 selectively binds to HR2, but not HR1 (Fig. 1e), implying that HR121 is functionally analogous to the HR1 trimer inner core in the fusion intermediate conformation, and could interact with its counterpart HR2. Using surface plasmon resonance (Biacore), we confirmed HR2 binding to HR121. The potent interaction between these two proteins had a dissociation constant (*K*_D_) of 0.686 nM, an association rate constant (*K*_a_) of 5.48 × 10^5^ M^−1^ s^−1^, and a dissociation rate constant (*K*_d_) of 3.76 × 10^−4^ s^−1^ (Fig. 1f). These values are comparable to the affinity between RBD and human ACE2 (hACE2).^7,13^ However, HR1 could not bind to HR121 at the concentration of 200 nM (data not shown). Circular dichroism (CD) spectrum also showed that HR121 exhibited a salient α-helix character, while HR2 alone showed a random structure, and HR1 alone showed a partly α-helix structure. When HR121 was mixed with either HR1 or HR2, it interacted with HR2 to form a more stable α-helix structure with typical double spectral minima at 208 nm and 222 nm. However, HR121 did not interact with HR1, and their mixture exhibited a less α-helical structure than that of HR121 (Fig. 1g). Together, our purified HR121 showed a high affinity to HR2, mostly mimicked the conformation, and resembled the function of the HR1 trimeric inner core in the fusion intermediate.

### HR121 induced highly potent cross-nAbs against SARS-CoV-2 in rabbits

**Fig. 2:**
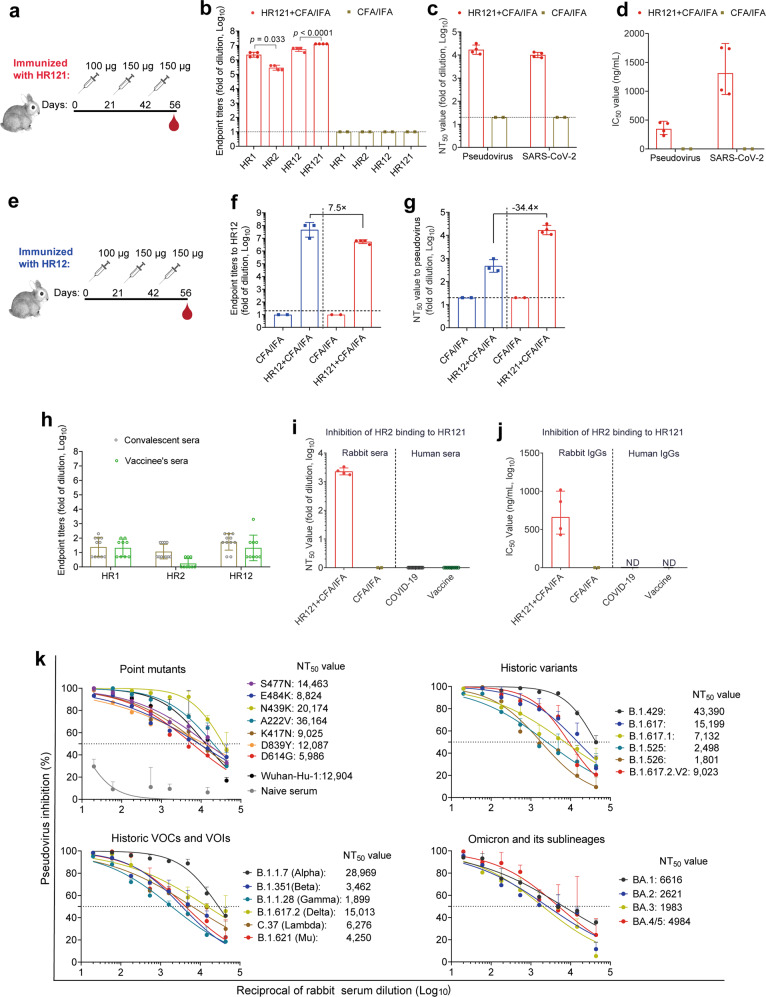
HR121 induced potent bAbs and nAbs in rabbits against SARS-CoV-2 variants. **a** Schematic diagram of rabbit immunization with HR121 formulated with CFA and IFA (*n* = 4) or CFA/IFA only (*n* = 2). **b** Endpoint titers of bAbs to purified SARS-CoV-2 HR proteins in HR121-immunized rabbits. *P* values were determined by ordinary one-way ANOVA. **c**, **d** nAbs to SARS-CoV-2 pseudovirus and authentic virus in HR121-immunized rabbits. nAbs in rabbit sera are presented with NT_50_s (**c**), and nAbs in IgGs purified from sera with IC_50_s (**d**). **e** Schematic diagram of rabbit immunization with HR12 with CFA/IFA (*n* = 3) or CFA/IFA only (*n* = 2). **f** Comparison of bAbs to HR12 protein between HR12 immunization and HR121 immunization. **g** Comparison of nAbs to SARS-CoV-2 pseudovirus between HR12 immunization and HR121 immunization. **h** bAbs to HR proteins in sera from human COVID-19 convalescent (*n* = 11) and vaccinated individuals (*n* = 9). **i**, **j** Inhibitions of HR121 and HR2 binding by sera (**i**) and IgGs (**j**) from HR121-immunized rabbits were determined by a competitive ELISA, and inhibitions by sera from human COVID-19 convalescents (*n* = 11) and vaccinated individuals (*n* = 9) were also evaluated. ND, not determined. **k** Dose-dependent curves of rabbit anti-HR121 sera to 23 current SARS-CoV-2 pseudo-variants. The NT_50_s are marked (dashed lines) and calculated by GraphPad Prism 8.0.1. In **b**–**d**, **f**, **g**, and **h**–**j**, data are presented as geometric mean ± geometric standard deviation (SD), and horizontal dashed lines mean the limit of quantification.

To assess the immunogenicity of HR121, we formulated HR121 with Freund’s adjuvant, and subcutaneously injected the combination into four rabbits three times at three-week intervals. Complete Freund’s adjuvant (CFA) was used in the prime immunization, and incomplete Freund’s adjuvant (IFA) was used in the two boosts (the same procedure was used in the mouse and hamster immunizations). Sera were collected two weeks after the last immunization. Two adjuvant-immunized rabbits were used as mock controls (Fig. 2a). We observed that HR121 evoked homogeneously high endpoint titers of autologous IgG antibodies (geometric mean: 1.3 × 10^7^), slightly lower geometric mean titers (GMTs) to 6-HB scaffold (5.4 × 10^6^) and HR1 (2.4 × 10^6^), and weak antibodies to HR2 (3.0 × 10^5^) in the rabbit sera (Fig. 2b). By serially diluting sera on cell culture plates, we observed that the nAbs evoked by HR121 were highly inhibitory, preventing VSV pseudotyped SARS-CoV-2 env (Wuhan-Hu-1 strain) entry into 293T-hACE2 cells and authentic SARS-CoV-2 prototype strain replication in human pulmonary alveolar epithelial cells (HPAEpiCs), with a geometric mean of 50% neutralization titer (NT_50_) = 1.7 × 10^4^ and 1.0 × 10^4^, respectively (Fig. 2c; Supplementary information, Fig. S3a, c). The corresponding IgGs purified from the sera also demonstrated high neutralizing activities against the pseudovirus and authentic SARS-CoV-2, with geometric mean half-maximal inhibitory concentration (IC_50_) of 346 ng/mL and 1312 ng/mL, respectively (Fig. 2d; Supplementary information, Fig. S3b, d).

To determine whether HR12 protein can also be used as a vaccine antigen to elicit nAbs against SARS-CoV-2, we immunized three rabbits with HR12 protein (the 6HB scaffold) using the same procedure as that for immunization with HR121 (Fig. 2e). Interestingly, we found that HR12 could induce higher endpoint titers of HR12-binding IgGs (geometric mean: 4.6 × 10^7^), ~7.5-fold higher than that of sera from HR121-immunized rabbits (geometric mean: 5.4 × 10^6^) (Fig. 2f). The antibodies evoked by HR12 showed low neutralizing activity against the prototype SARS-CoV-2 pseudovirus with a GMT value of 4.8 × 10^2^, which was 34.4-fold lower than that of HR121 (GMT: 1.7 × 10^4^) (Fig. 2g; Supplementary information, Fig. S3a). These results suggest that HR12 can elicit high titer HR12 binding antibodies (bAbs) with weak SARS-CoV-2 neutralizing activity and that HR12 protein is not a good vaccine antigen.

Among the antibodies to HR121, those targeting the two unoccupied surface grooves formed in the outer HR1 trimer of HR121 (Fig. 1d), thus inhibiting HR121 binding to HR2, would play the main antiviral roles. Using a competitive enzyme-linked immunosorbent assay (ELSIA) previously reported,^56^ we found that both rabbit anti-HR121 sera and IgG antibodies could potently inhibit HR2 binding to HR121, with geometric mean NT_50_ and IC_50_ at 2.3 × 10^3^ and 663 ng/mL, respectively (Fig. 2i, j; Supplementary information, Fig. S3e, f), which were consistent with their antiviral activities (Fig. 2c, d; Supplementary information, Fig. S3a–d). These results provide a proof of principle that the nAbs evoked by HR121 mainly target HR2 binding to the HR1 trimer, thus blocking membrane fusion and viral entry into host cells.

In human COVID-19 convalescent and vaccinated populations, rare nAbs targeting HR1 and HR2 domains in the S2 subunit were isolated.^33,57^ Here, we detected moderate levels of anti-HR1, -HR2, and -HR12 antibodies in the sera of these individuals (Fig. 2h), which is consistent with the results of a previous report.^58^ However, none of their sera could inhibit the binding between HR2 and HR121 in the competitive ELISA (Fig. 2i; Supplementary information, Fig. S3g, h), implying that few nAbs targeting membrane fusion could be generated from these populations. These results suggest that HR121 is a unique vaccine candidate that evokes nAbs targeting the HR1 domain in the S2 fusion intermediate, whereas none of the currently reported COVID-19 vaccines target the viral and cellular membrane fusion steps.

Because anti-HR121 sera and IgGs from the four rabbits showed similar activity against pseudotyped or authentic SARS-CoV-2, we pooled the sera and assessed their antiviral spectrum using a VSV pseudotyped SARS-CoV-2 spike system. The pooled sera exhibited high dose-dependent neutralizing activities against a series of SARS-CoV-2 variants, including 7 important point mutants (S477N, E484K, N439K, A222V, K417N, D839Y, and D614G) that decrease the efficacy of nAbs, and 12 historically pandemic variants generated from SARS-CoV-2-infected populations,^14,59^ as well as the current globally emerging Omicron BA.1 and its sublineages BA.2, BA.3, and BA.4/5 (BA.4 and BA.5 share the same spike).^25^ The NT_50_s of the rabbit sera ranged from 6.0 × 10^3^ to 3.6 × 10^4^ against the 7 point mutants and 1.8 × 10^3^ to 4.3 × 10^4^ against the 12 previous pandemic variants. Notably, the anti-serum potently neutralized the 6 main VOCs and variants of interest (VOIs), with NT_50_s of 2.9 × 10^4^ to B.1.1.7 (Alpha), 3.5 × 10^3^ to B.1.351 (Beta), 1.9 × 10^3^ to B.1.1.28 (Gamma), 1.5 × 10^4^ to B1.617.2 (Delta), 6.3 × 10^3^ to C.37 (Lambda), and 4.3 × 10^3^ to B.1.621 (Mu). The serum also neutralized the circulating variant B.1.1.529 (Omicron BA.1) at a titer of 6.6 × 10^3^, and its current sublineages BA.2, BA.3, and BA.4/5 at titers of 2.6 × 10^3^, 2.0 × 10^3^, and 5.0 × 10^3^, respectively (Fig. 2k). These data suggest that our HR121 is a promising immunogen that induces high nAb titers and exhibits broad activity against the main ancestral and current SARS-CoV-2 variants entering host cells.

Previous studies have demonstrated that the nAbs in the convalescent sera from SARS-CoV-2-infected hamsters^60^ or monkeys^61^ exhibited protective roles in these animals against subsequent viral rechallenge, and passive administration of the convalescent sera to naïve hamsters^60^ or monkeys^62^ effectively suppressed the viral replication in their lung tissues. To further evaluate the in vivo antiviral role of nAbs in the rabbit sera, we injected the purified IgG from the rabbit sera intraperitoneally into hACE2 transgenic mice and Syrian golden hamsters at a dose of 5 mg IgGs/20 g body weight, and estimated the viral genomic RNAs (gRNAs) and subgenomic RNAs (sgRNAs) in their lung tissues 3 days post SARS-CoV-2 challenge using real-time qRT-PCR assays. The gRNA is a marker of viral particles, and sgRNA is an indicator of viral replication.^7^ After injecting anti-HR121 IgGs, no gRNAs were detected in 6/6 mice and 7/9 hamsters. In the remaining 2 hamsters, the sgRNAs were not detectable (Supplementary information, Fig. S4), suggesting that the residual gRNAs in the lung tissues of these two hamsters were inactive. This result indicates that the nAbs evoked by HR121 can completely block SARS-CoV-2 replication in vivo.

### HR121 induced prolonged humoral and cellular immune responses in BALB/c mice

**Fig. 3:**
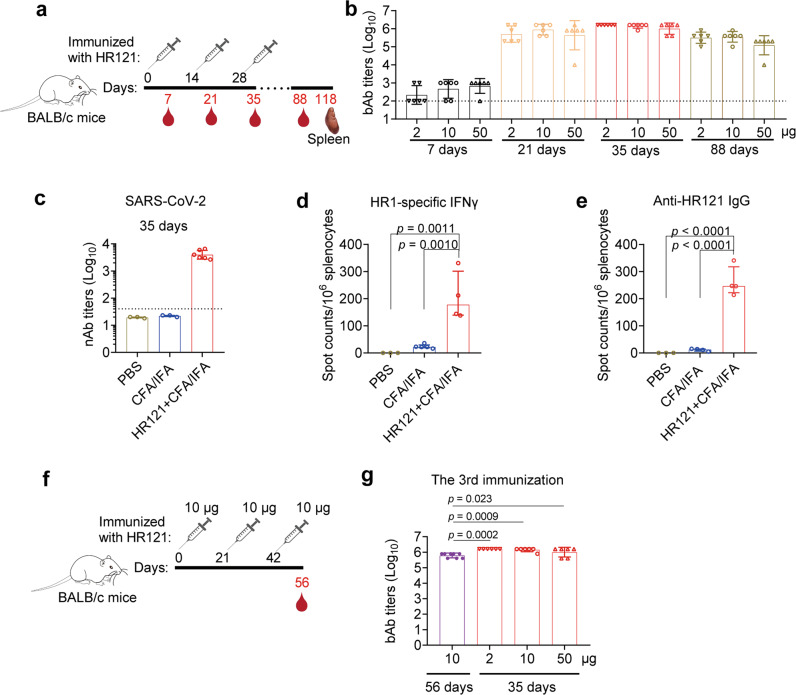
HR121 induced prolonged humoral and cellular immune responses in BALB/c mice. **a** Scheme of BALB/c mouse immunization with HR121/Freund’s adjuvant at two-week intervals. Three groups of mice were immunized with different doses of HR121 plus CFA/IFA, and another two groups of mice were injected with CFA/IFA and PBS as negative controls. *n* = 6 per group. **b** Endpoint titers of bAbs to HR121. In another two groups of mice injected with adjuvant and PBS, the bAbs were not detectable (data not shown). **c** nAbs to authentic SARS-CoV-2 in the group immunized with 10 μg of HR121 at 7 days after the third immunization. **d** HR1-specific cellular immunity at 90 days after the third immunization. **e** HR121-specific humoral immunity at 90 days after the third immunization. **f** Scheme of BALB/c mouse immunization with HR121/Freund’s adjuvant at three-week intervals. **g** Endpoint titers of bAbs to HR121 at three-week intervals (colored in purple) compared with those at two-week intervals (colored in red). In **b**–**e** and **g**, each symbol represents a mouse. The bAbs and nAbs against HR121 in the controls are expected to be negative; therefore, we selected some of them to test, and the same is applied to the other figures. In **b**, **c** and **g**, data are presented as geometric mean ± geometric SD, and dashed lines represent the limit of quantification. In **d** and **e**, data are presented as median ± interquartile range. In **b**, **d**, **e**, and **g**, differences between each group are determined by one-way ANOVA.

To evaluate antibody production, we subcutaneously injected three groups of BALB/c mice with 2 μg, 10 μg, and 50 μg of HR121 in Freund’s adjuvant (CFA/IFA), respectively. Immunizations were performed three times at two-week intervals, and sera were collected 7 days after each dose for antibody examination (Fig. 3a). Mice immunized with the adjuvant and PBS were used as controls. There were no remarkable differences in anti-HR121 IgG production among the administered HR121 doses. The first dose induced low HR121 IgG titers (GMTs ranging from 2.2 × 10^2^ to 6.8 × 10^2^); antibodies increased sharply after the second dose (GMTs ranging from 4.3 × 10^5^ to 9.0 × 10^5^), and peaked after the third dose (GMTs ranging from 1.0 × 10^6^ to 1.6 × 10^6^). Notably, even 2 months after the third immunization, HR121 IgG titers remained stable, only decreasing ~4-fold in the 2 μg and 10 μg dose groups, and ~8-fold in the 50 μg dose group (Fig. 3b).

We also evaluated nAb production by immunization with 10 μg dose of HR121 at a two-week interval. After the third immunization, the geometric mean NT_50_ against authentic SARS-CoV-2 reached 4.0 × 10^3^ (Fig. 3c). Three months after the third immunization, the mice in this group were euthanized and their splenocytes were isolated and stimulated with a pool of 15-amino-acid overlapped peptides covering the full HR1 sequence. In the HR121-immunized mice, an increased level of cytokine interferon gamma (IFNγ) was observed using enzyme-linked immune absorbent spot (ELISpot) assays (Fig. 3d). These data suggested that even after three months of the last immunization, these mice still maintained a strong cellular immune response. To evaluate the number of antibody-producing cells in the HR121-immunized mice, we cultured their splenocytes without stimulation and found an increased secretion of anti-HR121 IgGs even 90 days after the last immunization by ELISpot (Fig. 3e), which was consistent with the robust and sustained antibody responses detected in the sera, suggesting that the sustained plasma cell and memory B cell responses were elicited in the HR121-immunized mice.

We also immunized BALB/c mice with 10 μg of HR121 at three-week intervals (Fig. 3f), and observed antibodies 2.3-fold lower (GMT: 6.2 × 10^5^) than those generated at two-week intervals (Fig. 3g). Together, these data suggest that HR121 is a strong immunogen, consistent with the results of previously reported RBD-HR vaccines, in which the HR1 and HR2 were linked together as a 6-HB scaffold to enhance both humoral and cellular immune responses in the vaccine-immunized mice.^9,63^

### HR121 elicited high titers of nAbs against SARS-CoV-2 infection in hACE2 transgenic mice

**Fig. 4:**
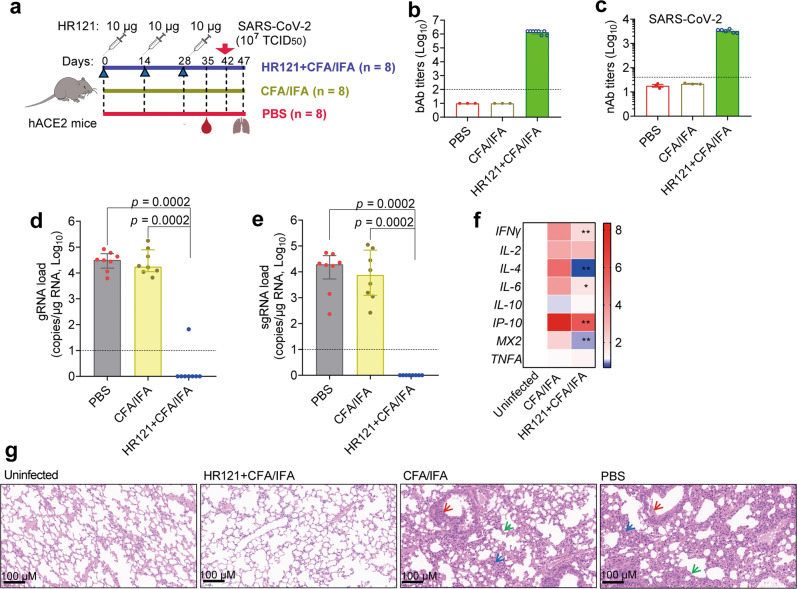
HR121 induced highly potent nAbs in hACE2 transgenic mice against SARS-CoV-2 infection. **a** Schematic illustration of hACE2 mouse vaccination with HR121 and challenge with SARS-CoV-2. **b** bAbs to HR121. **c** nAbs to authentic SARS-CoV-2. Two mice vaccinated with HR121 were not examined for their low volumes of sera. **d** Pulmonary viral gRNA loads. **e** Pulmonary sgRNA loads. **f** Heatmap of pulmonary cytokine expressions. RNA levels of antiviral and pro-inflammatory cytokine genes in the groups of adjuvant and HR121 (6/8 in each group) were normalized to those of uninfected controls (*n* = 3). Data are presented as median ± interquartile range. Differences between adjuvant and HR121 groups were determined by two-tailed Mann-Whitney test. *P < 0.05, **P < 0.01. **g** Representative photographs of H&E staining, in which infiltration of lymphocytes and macrophages (blue arrow), detached bronchial mucosa (red arrow) and thickened alveolar walls (green arrow) in PBS and adjuvant controls are marked. In **b**–**e**, each circle/dot represents a mouse, and dashed lines represent the limit of quantification. Data are presented as geometric mean ± geometric SD. In **d** and **e**, data are presented as median ± interquartile range (two-tailed Mann-Whitney test).

Thereafter, we selected a 10 μg dose of HR121 to vaccinate hACE2 transgenic mice. Using the same vaccination procedure as that in the BALB/c mouse experiment (Fig. 4a), we detected similar titers of HR121-binding IgGs (GMT: 1.3 × 10^6^) (Fig. 4b) and nAbs to authentic SARS-CoV-2 (GMT: 3.3 × 10^3^) (Fig. 4c). After being challenged with a high titer of SARS-CoV-2 (TCID_50_ = 10^7^), 7/8 mice were completely protected from the viral infection. One mouse had a few remnant virions in its lung tissue (Fig. 4d), but they were inactive as no viral sgRNAs were detected (Fig. 4e). Additionally, mice vaccinated with HR121 showed decreased levels of some inflammation-related cytokines (IL-4, IL-6 and IP-10) and antiviral cytokines (IFNγ and MX2) and their genes in the lung tissues, compared to those of the adjuvant controls (Fig. 4f; Supplementary information, Fig. S5c), implying that the inflammatory immune responses induced by SARS-CoV-2 infection were reduced in these mice. Hematoxylin and eosin (H&E) staining showed that mice vaccinated with HR121 did not have obvious histopathological changes in their lung tissues (Fig. 4g; Supplementary information, Fig. S5a). In contrast, in the two control groups (PBS and adjuvant alone), the lung tissues showed typical features of viral interstitial pneumonia, including infiltration of lymphocytes and macrophages, detached bronchial mucosa, and thickened alveolar walls (Fig. 4g). Immunohistochemistry staining also did not detect the nucleocapsid protein of SARS-CoV-2 in the lung tissues of HR121-vaccinated mice. By contrast, in PBS and adjuvant controls, the nucleocapsid protein was aggregated around the bronchial epithelial cells and alveolar epithelia (Supplementary information, Fig. S5b). Thus, these data suggest that HR121 elicits potent nAbs in hACE2 transgenic mice against SARS-CoV-2 infection.

### HR121 provided long-term protection against SARS-CoV-2 infection for Syrian golden hamsters

**Fig. 5:**
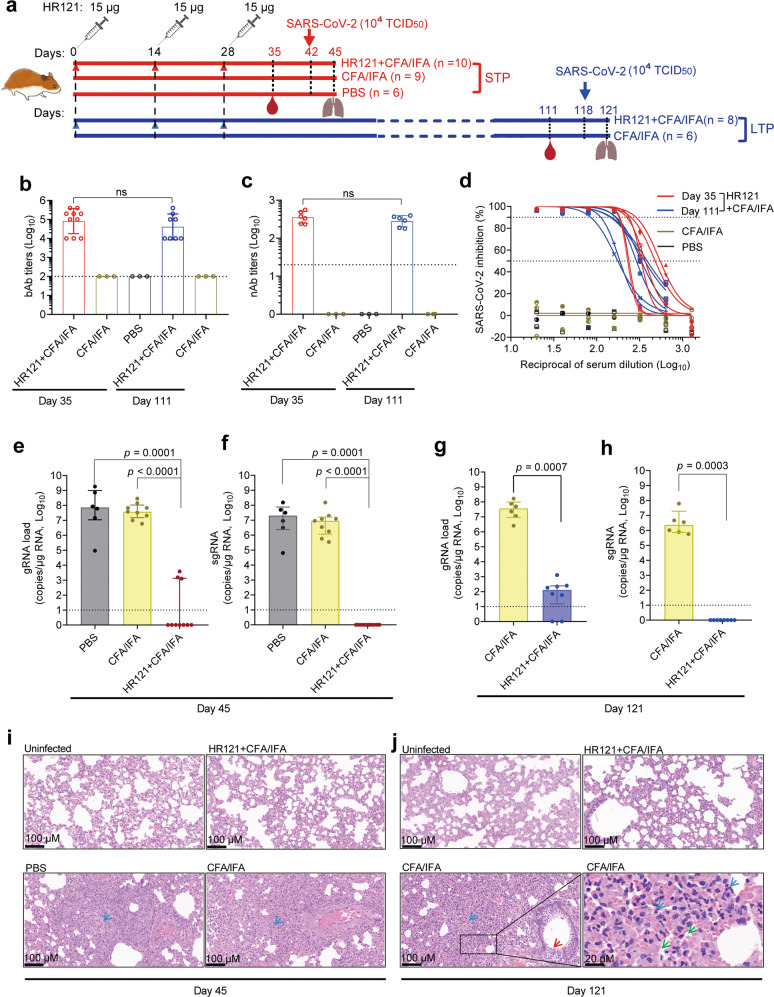
HR121 induced long-term protection in Syrian golden hamsters from SARS-CoV-2 infection. **a** Schematic illustration of vaccination of two groups of hamsters with HR121. They were divided into a STP group and a LTP group. **b** bAbs to HR121. **c** nAbs against authentic SARS-CoV-2. Four and two hamsters in the STP an LTP groups were not examined for the low volumes of sera obtained, respectively. **d** Dose-dependent curve of nAbs to authentic SARS-CoV-2. **e**, **f** Pulmonary viral gRNA (**e**) and sgRNA (**f**) in STP group. **g**, **h** Pulmonary viral gRNA (**g**) and sgRNA (**h**) in LTP group. **i**, **j** Representative photographs of pulmonary H&E staining in the STP group (**i**) and LTP group (**j**), in which infiltration of lymphocytes and macrophages (blue arrow), detached bronchial mucosa (red arrow), and diffuse alveolar damage (green arrow) in PBS or adjuvant controls are marked. In **b**, **c** and **e**–**h**, each circle/dot represents a hamster, and dashed lines mean limit of quantification. In **d**, each line represents a hamster. In **b** and **c**, data are presented as geometric mean ± geometric SD (two-tailed Mann-Whitney test). In **e**–**h**, data are presented as median ± interquartile range (two-tailed Mann-Whitney test).

We further assessed the protective efficiency of HR121 in Syrian golden hamsters. Two groups of hamsters were subcutaneously vaccinated with 3 doses of HR121 (15 μg each) formulated with Freund’s adjuvant (CFA/IFA) at two-week intervals. Then, they were separated into short-term protective (STP) and long-term protective (LTP) groups. The SARS-CoV-2 (TCID_50_ = 10^4^) challenge was carried out two weeks or three months after the last vaccination in the STP and LTP groups, respectively (Fig. 5a). We observed that HR121 induced a relatively moderate humoral immune response in hamsters compared with that in mice. The anti-HR121 IgG titers reached a geometric mean of 8.1 × 10^4^ after the third HR121 dose, with no obvious attenuation three months after the last immunization (Fig. 5b). Correspondingly, the GMT of nAbs to authentic SARS-CoV-2 in the STP group was 3.5 × 10^2^ and was similar in the LTP group (Fig. 5c). Although the nAbs were relatively lower than those generated in mice, the nAbs showed dose-dependent inhibition against SARS-CoV-2 replication in HPAEpiC cells (Fig. 5d).

The lung tissues of hamsters were collected on day 3 after the viral challenge. In the STP group, only 3/10 HR121-vaccinated hamsters had detectable viral gRNAs (Fig. 5e), most likely from the remnant inactive viral genomes, since no viral sgRNAs were determined (Fig. 5f). In the LTP group, even three months after the vaccination, HR121 showed efficient protection in hamsters. Viral gRNAs were detected in 6/8 hamsters with ~5 Log reduction (median), compared to those in adjuvant controls (Fig. 5g). Again, these gRNAs should be inactive since no viral sgRNAs were detected (Fig. 5h). The lung tissues of hamsters in both STP and LTP groups showed no obvious pathological changes by H&E staining, similar to the healthy hamsters (Fig. 5i, j; Supplementary information, Fig. S6a). In the controls, pathological changes of the lung were observed, including severe infiltration of lymphocytes and macrophages, apparent thickened alveolar walls, diffuse alveolar damage, detached bronchial mucosa, and disappearance of recognizable architecture (Fig. 5i, j). The nucleocapsid protein staining also confirmed the uninfected states of HR121-vaccinated hamsters (Supplementary information, Fig. S6b). These results indicate that a relatively low level of nAb vaccination can provide long-term protection in hamsters against SARS-CoV-2 infection.

### HR121 elicited homogeneous nAbs against SARS-CoV-2 and its main variants in rhesus macaques

**Fig. 6:**
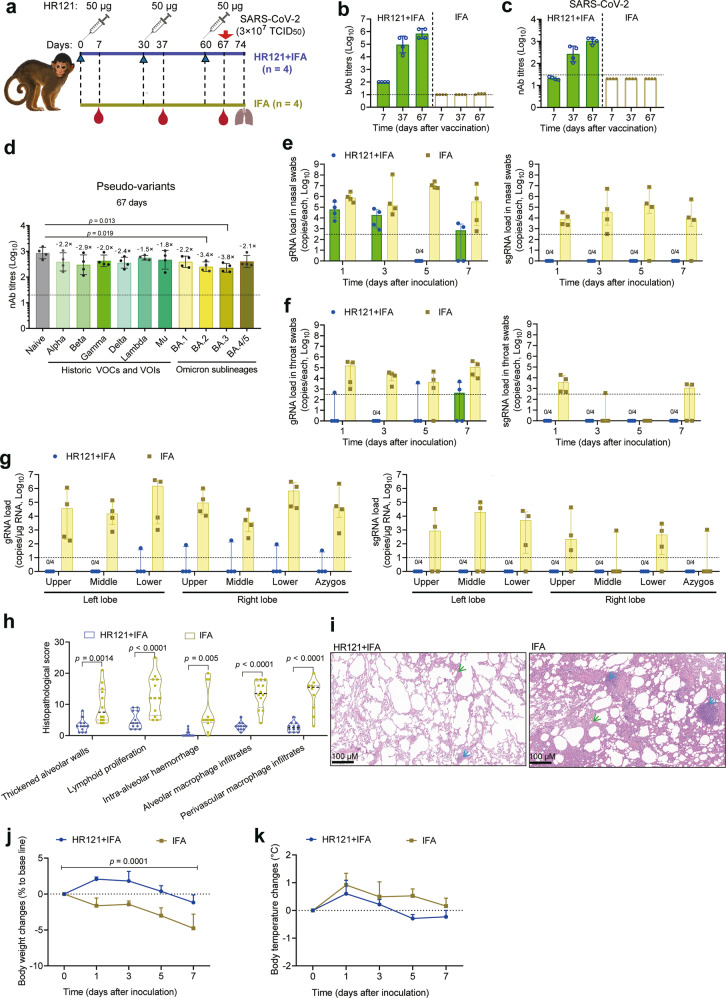
HR121 provided effective protection against SARS-CoV-2 infection in rhesus macaques. **a** Schematic diagram of vaccination of eight rhesus macaques with HR121 plus IFA (*n* = 4) and IFA only (*n* = 4). **b** bAbs to HR121. **c** nAbs to authentic SARS-CoV-2. **d** nAbs to VSV-based SARS-CoV-2 main circulating pseudo-variants. **e**–**g** Viral gRNA and sgRNA in nasal swabs (**e**), throat swabs (**f**), and lung lobes (**g**) of SARS-CoV-2-challenged macaques. **h** Histopathologic scores of lung lesions from HR121-vaccinated and adjuvant control macaques. Three representative samples from upper, middle, and lower lung lobes of each macaque were stained with H&E and evaluated. Nine regions of each sample were scored independently. Each lesion was assigned with a score of: 0, no substantial findings; 1, minimal; 2, mild; 3, severe. Scores were added for each lesion in one sample (Two-way ANOVA). **i** Representative photographs of pulmonary H&E staining in the HR121-vaccinated and adjuvant control macaques, in which thickened alveolar walls (green arrow), and lymphoid proliferation (blue arrow) are marked. Other lesions in lung tissues were labeled in Supplementary information, Fig. S8. **j** Body weight changes in SARS-CoV-2-challenged macaques. Data are presented as means ± SEM (Two-way ANOVA). **k** Body temperature changes in SARS-CoV-2-challenged macaques. Data are presented as means ± SEM (Two-way ANOVA). In **b**–**g**, each circle/dot represents a macaque, and dashed lines mean the limit of quantification. In **b**–**d**, data are presented as geometric mean ± geometric SD (two-tailed Wilcoxon test). In **e**–**g**, data are presented as median ± interquartile range (two-tailed Wilcoxon test).

To assess the efficacy of HR121 vaccine in nonhuman primates, we subcutaneously injected four rhesus macaques with 50 μg HR121 plus IFA, and another four rhesus macaques with IFA only as mock controls. Vaccinations were given three times at one-month intervals, and sera were collected seven days after each dose for antibody examination. The SARS-CoV-2 challenge was performed seven days after the last vaccination (Fig. 6a). We found that HR121 elicited a strong antibody response in rhesus macaques. The first dose induced low HR121-binding IgG titers with GMT of 1.0 × 10^2^; GMTs of the IgG titers increased to 9.5 × 10^4^ after the second dose, and peaked at 6.9 × 10^5^ after the third dose (Fig. 6b). Correspondingly, the nAbs to SARS-CoV-2 were undetectable after the first dose, increased to 2.7 × 10^2^ and 1.1 × 10^3^ after the second and third doses, respectively (Fig. 6c; Supplementary information, Fig. S7a). The cross-nAbs in the sera of the HR121-vaccinated macaques were evaluated after the third dose using the VSV pseudotyped viruses. Notably, HR121 induced broad-spectrum and homogeneous nAbs to the main circulating variants in these macaques. Compared with the naïve (Wuhan-Hu-1) strain, the GMTs of nAbs to the 6 previously circulating variants (Alpha, Beta, Gamma, Delta, Lambda, and Mu) only dropped by 1.5–2.9 folds, and no significant differences were observed among them (*P* > 0.05, ordinary one-way analysis of variance (ANOVA)). Encouragingly, the nAbs had a considerable effect against the current circulating variants of Omicron, with GMTs of 3.9 × 10^2^ to BA.1, 2.5 × 10^2^ to BA.2, 2.3 × 10^2^ to BA.3, and 4.0 × 10^2^ to BA.4/5. The titers of nAbs to BA.2 and BA.3 had a slight decrease by 3.4 and 3.8 folds, respectively, compared with that of the naïve strain. The nAbs demonstrated similar activities against Omicron and its sublineages, implying that the mutations in the sublineages of Omicron, which are mainly located in the S1 subunit of the S protein, are possibly unrelated to the efficiency of the membrane fusion and thus cannot dampen the efficacy of HR121-elicited nAbs (Fig. 6d; Supplementary information, Fig. S7b). Interestingly, the homogeneity of the nAbs evoked in macaques was slightly different from those in rabbits, which varied among different strains (Fig. 2k). This discrepancy may be due to the diverse Ig germline generations in different species and individuals.^64,65^ Generally, in both rabbits and macaques, HR121 induced potent cross-nAbs against the main previous and current variants of SARS-CoV-2.

### HR121 provided nearly full protection against SARS-CoV-2 infection in rhesus macaques

Taking into consideration the high levels of nAbs elicited by HR121 in macaques, which are similar to those elicited in BALB/c (Fig. 3c) and hACE2 transgenic mice (Fig. 4c), and the possibility of the undetectable viral RNAs in some pulmonary tissues of SARS-CoV-2-challenged macaques as previously reported,^66,67^ we challenged these eight macaques with a high titer of SARS-CoV-2 (TCID_50_ = 3 × 10^7^) equally through intranasal and intratracheal routes. Subsequently, we collected nasal and throat swabs on days 1, 3, 5, and 7 after the challenge. In adjuvant-injected macaques, viral gRNAs were consistently detectable, and remained at high levels in nasal swabs (medians ranged from 1.1 × 10^6^ to 2.8 × 10^7^ copies per test) and throat swabs (medians ranged from 1.3 × 10^4^ to 1.6 × 10^5^ copies per test) at each sampling time point. In HR121-immunized macaques, viral gRNAs were only detected at a relatively high level in nasal swabs on day 1 (median: 1.2 × 10^5^ copies per test), with a decline on day 3 (median: 2.8 × 10^4^ copies per test) after the challenge. The gRNAs were occasionally detectable in throat swabs. Accordingly, it can be inferred that these viral gRNAs were from remnant inactive virions or from the remaining inoculations, as no viral sgRNAs in HR121-immunized macaques were detected in both the nasal and throat swabs at all sampling time points (Fig. 6e, f).

On day 7 after the challenge, the macaques were euthanized and their lung tissues were collected for viral load evaluation and pathological analysis. In adjuvant-immunized macaques, the viral gRNAs (medians ranged from 9.3 × 10^3^ to 1.8 × 10^6^ copies/μg RNA) remained at high levels in all tested lung lobes, showing a similar pattern to those in nasal and throat swabs. Likewise, few viral gRNA remnants were detected in only one HR121-immunized macaque, and they were undetectable in later viral sgRNA tests (Fig. 6g). After H&E staining, severe pathological lesions were observed in adjuvant-injected macaques; the typical pathological damages included thickened alveolar walls, lymphoid proliferation, intra-alveolar hemorrhage, alveolar macrophage infiltrates, and perivascular macrophage infiltrates. However, these pathological lesions were minimal and occasionally revealed in HR121-immunized macaques (Fig. 6h, i; Supplementary information, Fig. S8), which coincided with the reported results of some SARS-CoV-2 vaccines.^7,68^ The observed pathological changes in the HR121-immunized macaques may be highly attributed to the acute immune responses following the high inoculation of viral particles into the trachea of these macaques. In HR121-immunized macaques, there were no considerable changes in body weight and body temperature after the viral challenge, whereas in the adjutant-injected macaques, the average body weight decreased by ~5% seven days (at the endpoint) after the challenge (Fig. 6j), and the average body temperature was a little higher than that of HR121-immunized macaques at all monitoring time points (Fig. 6k). These clinical signs observed suggest that HR121 has a protective role in rhesus macaques.

### HR121 offered effective protection against Omicron BA.2 variant infection in Syrian golden hamsters

**Fig. 7:**
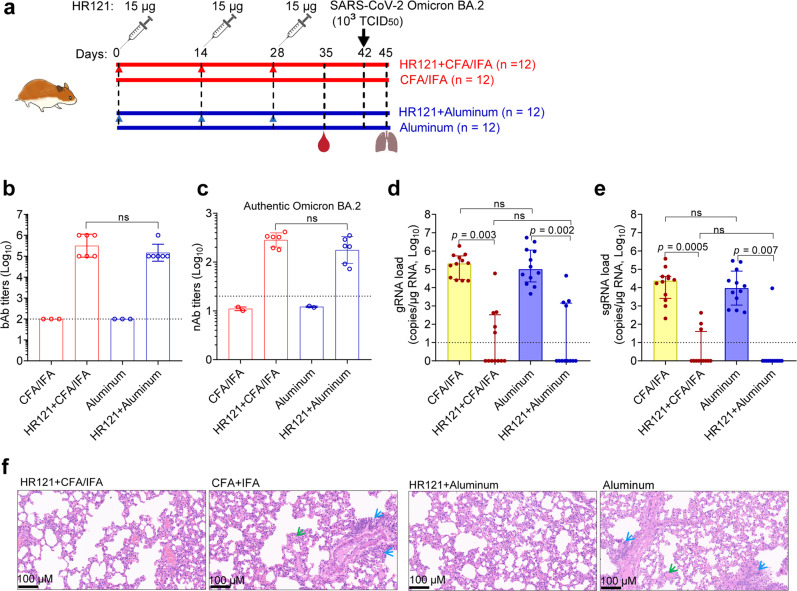
Vaccination with HR121 formulated with aluminum or Freund’s adjuvant (CFA/IFA) resulted in similarly effective protection of Syrian golden hamsters against challenge with SARS-CoV-2 Omicron BA.2 variant. **a** Schematic illustration of injection of four groups of hamsters (*n* = 12 per group) with HR121 plus CFA/IFA (Group 1), CFA/IFA only (Group 2), HR121 plus aluminum adjuvant (Group 3), and aluminum adjuvant only (Group 4). **b** bAbs to HR121. **c** nAbs against authentic SARS-CoV-2 Omicron BA.2 variant. **d**, **e** Pulmonary viral gRNA (**d**) and sgRNA (**e**) in the four groups. **f** Representative photographs of pulmonary H&E staining in the four groups, in which infiltration of lymphocytes and macrophages (blue arrow), and alveolar damage (green arrow) in the adjuvant only controls are marked. In **b**–**e**, each circle/dot represents a hamster; in **b** and **c**, the sera of two hamsters in each HR121 vaccination were pooled because of their low volumes. In **b**–**e**, dashed lines mean limit of quantification. In **b** and **c**, data are presented as geometric mean ± geometric SD (two-tailed Wilcoxon test). In **d** and **e**, data are presented as median ± interquartile range (two-tailed Wilcoxon test).

Finally, we assessed the in vivo efficacy of HR121 vaccine against the currently emerging SARS-CoV-2 Omicron variant BA.2. One group of hamsters were subcutaneously vaccinated with HR121 formulated with Freund’s adjuvant (CFA/IFA) (Freund’s adjuvant only as control), while another group of hamsters were intramuscularly vaccinated with HR121 formulated with a clinical-grade aluminum adjuvant (aluminum adjuvant only as control). Both vaccinations were administered with the same dose of HR121 (15 μg each) three times, at two-week intervals. Omicron BA.2 (TCID_50_ = 10^3^) challenge was carried out two weeks after the last vaccination (Fig. 7a). After the vaccination, we found that HR121/aluminum adjuvant could elicit HR121 bAbs (GMT: 1.5 × 10^5^) at the similar level to that induced by HR121/Freund’s adjuvant (GMT: 3.2 × 10^5^) (Fig. 7b). The titers of nAbs against authentic Omicron BA.2 in the sera from these two groups of hamsters were also at a similar level, with GMT values of 2.8 × 10^2^ in the HR121/Freund’s adjuvant group and 1.8 × 10^2^ in the HR121/aluminum adjuvant group, respectively (Fig. 7c).

At 3 days post the Omicron BA.2 challenge, the viral loads and pathological changes in lung tissues of these hamsters were examined. The pulmonary gRNAs in both Freund’s adjuvant and aluminum adjuvant controls were detected with median values of 2.1 × 10^5^ and 1.0 × 10^5^, respectively. There were no significant differences between them. gRNAs were detected in 5/12 hamsters in the HR121/Freund’s adjuvant group and 4/12 hamsters in the HR121/aluminum adjuvant group, with 5.3 and 5.0 Log reductions of the median values of the total numbers (12/12), respectively (Fig. 7d). Correspondingly, the pulmonary sgRNAs in both Freund’s and aluminum adjuvant controls were detected with median values of 5.8 × 10^4^ and 5.9 × 10^4^, respectively (no significant differences). The sgRNAs were detected in 3/12 and 1/12 hamsters in the HR121/Freund’s adjuvant and the HR121/aluminum adjuvant groups, with 4.4 and 4.0 Log reductions in the median values of the total numbers, respectively (Fig. 7e). After the H&E staining of the lung tissues, no apparent pathological changes were observed in both the HR121/Freund’s adjuvant and HR121/aluminum adjuvant groups, whereas in the Freund’s or aluminum adjuvant controls, slight to moderate degrees of infiltration of lymphocytes and macrophages, along with alveolar damage were presented (Fig. 7f). Together, these results suggest that HR121 formulated with Freund’s or aluminum adjuvants could induce similarly effective protection against Omicron BA.2 infection in the vaccinated Syrian golden hamsters.

## Discussion

The mechanisms of viral and host cellular membrane fusion are intriguing. Efforts have been made to design mimetics of the HR1 domain present in the fusion intermediate conformation, such as various HR1 trimers or 5-Helix, as fusion inhibitors against HIV-1,^47,69,70^ and SARS-CoV-2.^71^ Other research groups have applied the mimetics of the HR1 domain in the HIV-1 gp41 to design antibody-based HIV-1 vaccines,^48,51,52^ but these studies were terminated due to the poorly elicited nAbs. To date, there has been no report showing that the mimetics of HR1 domain present in the fusion intermediate conformation from a coronavirus is applied to vaccine design, although some groups have used the fragment consisting of HR1 and HR2 in SARS-CoV-2 S2 subunit for its conjugation with RBD to design RBD-HR-based vaccines.^9,63^ In this study, we designed a recombinant protein HR121, which showed a stable structure with a partially exposed HR1 trimeric structure, in which two surface grooves remained unoccupied, analogous to the theoretical HR1 trimeric inner core in the fusion intermediate conformation of the S2 subunit. As a proof of principle, HR121 is able to induce high titers of nAbs against SARS-CoV-2 in the immunized animals, such as rabbits, BALB/c mice, hACE2 transgenic mice, Syrian golden hamsters, and rhesus macaques. The high nAb titers evoked by HR121 from SARS-CoV-2 were different from those induced by other fusion intermediate mimetics from HIV-1.^48,51,52^ The reason for this discrepancy is unknown but may be related to: (1) differences of the efficient antigenic epitopes in different immunogens; the full HR1 and HR2 sequence in HR121 may improve its immunogenicity, and the two exposed surface grooves in one of HR1 trimeric structure of HR121 may provide more efficient antigenic epitopes for induction of nAbs; (2) differences of steric hindrances in the fusion intermediates; the fusion intermediate structure in HIV-1 was estimated with a length of 100 Å, which has provided enough space for IgGs to enter their binding sites,^72^ whereas in SARS-CoV-2, with an unusually prolonged HR1 domain, the length of the fusion intermediate complex was calculated to be at least 180 Å,^6,28^ providing a larger space for IgG accession.

In other studies, the serum nAb titers have been proved a main immune correlate of protection in evaluating the efficacies of COVID-19 vaccines.^12,73,74^ Here, we also demonstrated that the potent nAb responses induced by HR121 could provide nearly full protection against SARS-CoV-2 infection in several SARS-CoV-2-susceptible animal models, including hACE2 transgenic mice, Syrian golden hamsters and rhesus macaques. Aside from the robust antibody responses, we did not observe the HR121-elicited antibody-dependent enhancement of viral infection in either in vitro or in vivo experiments, in line with the result of the RBD-HR-based nanoparticle vaccine in which HR1 and HR2 domains were linked together as a scaffold and immunoenhancer.^9^ Thus, these results suggest that HR121 is a promising COVID-19 vaccine candidate.

Currently, the continuous emergence and rapid evolution of Omicron sublineages including BA.1, BA.2 and BA.3 sublineages had showed widespread escapes from the neutralization by most antibodies and vaccines.^24,75–77^ Besides this, the recently evolved Omicron sublineages BA.4 and BA.5 demonstrated stronger immune evasion against the plasma of 3-dose vaccinees, and BA.1 or BA.2 convalescents.^25,78,79^ These two sublineages BA.4 and BA.5 have emerged and dominated in South Africa, and are rising worldwide.^3^ Apart from them, the ongoing COVID-19 pandemic and concomitantly continuous evolution of SARS-CoV-2 will constantly evoke more newly mutated or recombinant variants. Therefore, those vaccine strategies, such as updating the spike antigens, or vaccine boosters, may be cumbersome and inaccurate to match the speed of antigenic drift, which is similar to the current status of seasonal influenza vaccines. In this study, we demonstrated another strategy to design a novel spike antigen HR121, targeting the conserved HR1 domain of S2 subunit. The nAbs elicited in both HR121-vaccinated rabbits and rhesus macaques exhibited broad-spectrum neutralizing activity against the main previous and current SARS-CoV-2 variants, particularly the current globally circulating variant Omicron and its sublineages. This result coincides with the observation that most of the mutations among the SARS-CoV-2 variants were located in the S1 subunit,^1,14^ and provides an alternative target for the development of the next-generation COVID-19 vaccine. The design strategy of the recombinant protein vaccine HR121 may also be applied to other COVID-19 vaccine platforms, such as viral vector-based vaccines, virus-like particles and DNA/mRNA vaccines, and combined with other targeted COVID-19 vaccines, such as RBD vaccines and inactive viral vaccines.

In addition, several broadly neutralizing antibodies (bnAbs) targeting the conserved regions in S2 subunit, including the FP and stem helix (SH) in the region between HR1 and HR2, have been isolated from SARS-CoV-2-infected or vaccinated individuals. They all act at the membrane fusion steps and show cross-binding and neutralizing activities against sarbecoviruses,^31,33,80–84^ implying that fusion mechanism of S2 subunit is a promising target for broad-spectrum vaccine designs. However, there has been no report so far about the clinical or per-clinical studies on SARS-CoV-2 vaccines targeting the S2 subunit, although Ng et al. has reported that a DNA vaccine containing genes encoding membrane-bound SARS-CoV-2 S2 subunit can induce bnAbs against sarbecoviruses, while the bacterially produced recombinant S2 protein cannot elicit bnAbs.^85^ These results, together with those from our study, suggest that these S2-specific bnAbs isolated from the SARS-CoV-2-infected or vaccinated individuals may be elicited by the neutralizing epitopes in the conserved regions of S2 subunit at a special fusion conformation (e.g., pre-fusion, fusion intermediate or post-fusion conformation), while our HR121 vaccine can induce potent bnAb responses in the vaccinated animals, possibly because HR121 vaccine contains the transiently exposed neutralizing epitopes in the HR1 region of S2 subunit present in the fusion intermediate conformation. Therefore, it is both a great opportunity and a challenge to design an effective S2 subunit vaccine containing the exposed neutralizing epitopes in the conserved regions of S2 subunit at a proper conformation that can induce potent and broad-spectrum cross-nAb responses. Meanwhile, the approach taken herein provides a specific immunogen to evoke HR domain-targeted nAbs. This means that further isolation of the mAbs from mice immunized with HR121 is feasible, thus making the resultant humanized antibody constructed from mouse antibodies for SARS-CoV-2/COVID-19 therapy promising.

Finally, HR domains in S2 subunit are extraordinarily conserved among coronaviruses.^9,33^ Further, other class I enveloped viruses, including some of the most studied viral families, such as retroviruses, orthomyxoviruses, paramyxoviruses, filoviruses, and arenaviruses, share a similar membrane fusion mechanism, in which HR1 and HR2 participate.^35,36^ Therefore, our strategy for vaccine design may extend or optimize HR121 for better antiviral activity against some types of these viruses, with careful consideration for the accessibility of IgGs to the binding sites formed by fusion intermediates and binding valences of nAbs to viruses.

In summary, this study demonstrates that HR121 can mimic the HR1 domain present in the fusion intermediate conformation of the S2 subunit, and induce highly potent broad-spectrum antibodies against SARS-CoV-2 and its main variants. Thus, it provides another important target for the development of novel COVID-19 vaccines.

## Limitations of the study

There are several limitations in this study. First, we are unable to solve the structure of HR121/HR2 complex, or HR121/antibody complex. Particularly, the conformation of the target sites in the HR1 part of HR121 may change significantly after HR121 binding to HR2 or antibodies, it is difficult for us to show the critical neutralizing epitopes and HR2-binding sites in HR121. Second, the homogeneous antibody and nAb responses elicited in HR121-immunized animals, and the small size of animal numbers, especially rabbits and rhesus macaques, limited the performances of the correlation between the inhibition of HR121 binding with HR2 and nAbs, or the relationship of antibodies and nAbs. Third, with the outcomes of the undetectable viral sgRNAs in different SARS-CoV-2-challanged animals, we did not measure the anamnestic nAb titers after infection, as they had showed little to no changes compared to the nAb titers evoked by pre-challenge of some SARS-CoV-2 vaccines with nearly full protection.^66,86^ Finally, we mainly focused on the evaluation of the vaccine-elicited nAb responses and the protective outcomes in different HR121-immunized animals, while the cellular immune correlates of protection, particularly the roles of CD4^+^ T and CD8^+^ T cell responses, which are likely to participate in the control of viral infection and disease severity,^87^ were not well investigated.

## Materials and methods

### Ethics statements

The Ethics Review Board of the Kunming Institute of Zoology, Chinese Academy of Sciences (CAS) approved this study. All animal experiments were approved by the Ethics Committee of Kunming Institute of Zoology, CAS (assurance No.: SMKX-2021-01-006), and were carried out in strict compliance with the guidelines and regulations of the Animal Care and Use Committee, Kunming Institute of Zoology, CAS. All SARS-CoV-2 infection experiments were approved (assurance No.: KIZP3-XMZR-2021-05) and carried out in the biosafety level-3 (BSL-3) laboratory of Kunming Institute of Zoology, CAS. The human serum samples in this study were collected in accordance with the Declaration of Helsinki. Informed consent was obtained from each participant.

### Viruses and cells

The prototype SARS-CoV-2 strain (Accession No.: NMDCN0000HUI in the China National Microbiology Data Center (NMDC)) was provided by the Guangdong Provincial Center for Disease Control and Prevention (Guangzhou, China). The SARS-CoV-2 Omicron BA.2 variant was isolated from a patient in Yunnan Provincial Infectious Disease Hospital, China. Its whole genome was sequenced by researchers at BSL-3 laboratory of Kunming Institute of Zoology, CAS, Kunming, China, and submitted to NMDC (No. SUB1663744906574). The viruses were propagated in African green monkey kidney epithelial cells (Vero-E6) (ATCC, #1586) and titrated. Virions from the 4th passage were used in the current experiments. VSV-G pseudotyped virus (G*ΔG-VSV-Rluc) was kindly provided by Prof. Geng-Fu Xiao (Wuhan Institute of Virology, CAS). HEK293T cells were obtained from ATCC. 293T-ACE2 cells were provided by Prof. Yuxian He (Institute of Pathogen Biology, Chinese Academy of Medical Sciences). These cells were cultured in basal DMEM (Gibco, Beijing, China) supplemented with 10% FBS (Gibco, New Zealand). Human pulmonary alveolar epithelial cells (HPAEpiCs) were purchased from the ScienCell Research Laboratory (San Diego, CA, USA) and were cultured in basal DMEM supplemented with 10% FBS,^88^ and passages 6–8 were used in this study.

### Human serum samples

Nine serum samples from SARS-CoV-2-vaccinated participants and one healthy unvaccinated serum sample were collected from our laboratory. The participants were injected with two doses of inactivated SARS-CoV-2 vaccine (Sinovac Biotech, Beijing, China) within 1–2 months. Eleven serum samples from COVID-19 convalescent individuals were collected at Yunnan Provincial Infectious Disease Hospital. All convalescent patients had recovered from COVID-19 for 1–2 months.

### Animals

BALB/c mice and New Zealand white rabbits were purchased from the Experimental Animal Center of Kunming Medical University, China. The hACE2 transgenic mice were established as we previously reported.^89,90^ Syrian golden hamsters were purchased from Charles River Company (Beijing, China). Rhesus macaques were purchased from Kunming Primate Research Center, Kunming Institute of Zoology.

### Plasmid construction

Recombinant protein HR121 from the S protein of SARS-CoV-2 was designed as HR1–linker1–HR2–Linker2–HR1. Genes encoding the full amino acid sequence of HR1 (residues 912–988) and HR2 (residues 1163–1206) were derived from the S protein of SARS-CoV-2 isolate Wuhan-Hu-1 (accession No.: NC_045512).^91^ The nucleotide sequence encoding linker 1 (GSSGG) was GGAGGAAGCGGAGGA and the nucleotide sequence encoding linker 2 (SGGRGG) was AGCGGAGGAAGAGGAGGA. The gene encoding HR121 was synthesized and cloned into the *E. coli* expression vector pMCSG7 with an N-terminal SUMO tag using the ligation-independent cloning method.^92^

To express the GST-HR121 fusion protein, the full HR121 sequence was inserted into the *E. coli* expression vector, pGEX-6P-1, at the restriction enzyme sites of *Eco*RI and *Xho*I.

To express HR1, HR2, and HR12 proteins, the genes encoding these proteins were separately cloned into the *E. coli* expression vector, pET-30a, at *Eco*RI and *Xho*I restriction enzyme sites. Therefore, each of these expressed proteins contains the redundant 52 amino acid residues from the vector.

### Protein expression and purification

SUMO-tagged HR121 in the pMCSG7 vector was expressed in *E. coli* BL21 (DE3), and bacteria harboring the expression vector were grown at 37 °C in LB media supplemented with 100 μg/mL ampicillin. Protein expression was induced with 0.5 mM IPTG when the cells reached an optical density of 0.6 at 600 nm. The cells were then cultured at 16 °C for another 16 h. Then, the cells were harvested by centrifugation at 5000× *g* for 10 min at 4 °C. The cells were resuspended in lysis buffer (25 mM Tris-HCl, pH 8.0, 150 mM NaCl, 5 mM β-ME), and lysed using ultrasonication. Then, the supernatant containing the recombinant proteins was separated by centrifugation at 12,000× *g* for 30 min at 4 °C. The fusion proteins were isolated by Ni Sepharose 6 FF (GE Healthcare, Beijing, China), and the SUMO tag was removed by TEV enzyme (1:100 w/w) cleavage. The target protein was loaded to a Superdex 200 increase column (GE Healthcare) with buffer containing 25 mM Tris-HCl, pH 8.0, 150 mM NaCl and 2 mM DTT. Peak fractions containing HR121 dimer were pooled, concentrated to 25 mg/mL and stored in a –80 °C freezer.

The recombinant proteins HR1, HR2, and HR12 were expressed in *E. coli* BL21 (DE3) cells. Bacteria were induced with 1 mM IPTG for 12 h at 20 °C before harvesting by centrifugation. The collections were lysed in PBS buffer by ultrasonication. The supernatants were separated by centrifugation at 12,000× *g* for 30 min at 4 °C, and the target proteins were purified using Ni Sepharose 6 FF affinity column. Briefly, the column was washed in gradients with 20 mM and 50 mM imidazole. Proteins were then eluted by 100 mM imidazole and concentrated to 10 mg/mL in PBS through a concentrating column with a 3 kDa cutoff (Millipore, Bedford, MA, USA).

The fusion protein GST-HR121 was expressed and induced using the same method as that for the recombinant proteins HR1, HR2, and HR12. Purification was carried out by glutathione–Sepharose 4B affinity column (GE Healthcare).

### Crystallization and structure determination

Crystals were obtained using the sitting-drop vapor diffusion method by commercial crystallization kits and incubation at 16 °C for 10 days. The crystals appeared in a solution containing 0.2 M sodium fluoride and 20% (w/v) polyethylene glycol 3350. Then, the crystals were harvested using 20% ethylene glycol (v/v) as a cryoprotectant before flash freezing in liquid nitrogen. The crystals were analyzed with beamline BL02U1 at the Shanghai Synchrotron Radiation Facility.^93^ The structure was determined by the molecular replacement method using PHASER^94^ and refined using PHENIX.^95^ The structure of 6-HB of SARS-CoV-2 (Protein Data Bank: https://www.rcsb.org/, with the accession number 6LXT) was used as the initial search model. The model of HR121 was manually adjusted in COOT^96^ and refined to a resolution of 2.41 Å with *R*_work_ and *R*_free_ values of 24.9% and 29.1%, respectively. Details of data collection and refinement statistics are provided in Supplementary information, Table S1. All figures representing structures were prepared using PyMOL (http://pymol.org).

### GST pull-down assay

Excess HR1 or HR2 in the bacterial supernatants was mixed with glutathione–Sepharose 4B affinity gel containing GST-HR121. Blank glutathione–Sepharose 4B affinity gel mixed with HR1 or HR2 and glutathione–Sepharose 4B affinity gel containing GST-HR121 were used as negative controls. The mixture was incubated for 30 min at room temperature with gentle agitation. Then, the supernatants were removed by centrifugation at 500× *g* for 5 min. The gels were washed three times with PBS by the same method of centrifugation, and collected for 10% SDS-PAGE analysis.

### Surface plasmon resonance assay

HR121 binding to HR2 or HR1 was determined by surface plasmon resonance using a BIAcore 3000 instrument (GE Healthcare). Briefly, HR121 was immobilized on the flow cell of a CM5 sensor. HR2 or HR1 protein (12.5 nM, 25 nM, 50 nM, 100 nM, and 200 nM) was injected to run across the chip. A separate channel was set as a control. The binding assays were performed at 25 °C. HR2 or HR1 was dissolved in BIAcore running buffer and injected at a constant flow rate of 35 μL/min for 3 min. Dissociation data were collected for 10 min. The kinetic parameters were obtained using an automated program.

### CD spectroscopy

CD spectra were recorded using a Jasco spectropolarimeter (model J-815). 1 μM each of HR121 and HR2 or HR1 was dissolved in PBS. Using a 0.1 cm pathlength cuvette, wavelength spectra were recorded with a 1-nM step size and 1-nM bandwidth from 195 nm to 260 nm at 20 °C. The spectra were corrected by subtracting the solvent blank, PBS.

### Animal immunization

Rabbits, mice, and hamsters were subcutaneously immunized with HR121 or HR12 formulated with CFA (Sigma-Aldrich, Saint Louis, MI, USA) and IFA (Sigma-Aldrich). Macaques were subcutaneously immunized with HR121 formulated with IFA. Another group of hamsters were intramuscularly immunized with HR121 formulated with aluminum adjuvant (Alhydrogel^®^ adjuvant 2%, Invivogen, San Diego, CA, USA). All immunizations were performed in a prime boost-reboost manner.

For rabbit immunization, 11 adult female New Zealand White Rabbits (average weight: 2.8 kg) were used. Four and three rabbits were injected with HR121 plus CFA/IFA and HR12 plus CFA/IFA in the same procedure, respectively. Each rabbit was immunized with 100 μg protein on day 0, and then 150 μg protein on days 21 and 42. The other 4 rabbits were injected with equal volumes of Freund’s adjuvant as mock controls. Serum samples were collected 14 days after the third immunization. IgGs were purified from the serum samples using Protein A (BBI, Shanghai, China).

For BALB/c mouse (male, 8 weeks old) immunization, three groups of mice (*n* = 6 per group) were injected with 2 μg, 10 μg, or 50 μg HR121 plus CFA/IFA at 14-day intervals. Two groups of mice (*n* = 6 per group) were injected with equal volumes of CFA/IFA or PBS (mock controls). Serum samples were collected at 7 days post each immunization, and 60 days post the third immunization. At 90 days post the third immunization, the mice were euthanized, and their splenocytes were isolated as previously reported.^9^ To optimize the HR121 immunization interval in mice, another group of mice (*n* = 8) was injected with 10 μg HR121 at 21-day intervals. Serum samples were collected 7 days after the third immunization.

For hACE2 mouse (male, 8 weeks old) vaccination, 8 hACE2 mice were vaccinated with 10 μg HR121 plus CFA/IFA at 14-day intervals. Equal numbers of mice injected with Freund’s adjuvant or PBS were used as controls. Serum samples were collected 7 days after the third immunization. SARS-CoV-2 challenge was carried out 14 days after the third immunization.

Syrian golden hamsters (male, 8 weeks old) were injected with 15 μg HR121 formulated with adjuvant, or adjuvant, or PBS only at 14-day intervals. For SARS-CoV-2 challenge studies, 39 hamsters were divided into two groups for short- and long-term protection studies. In STP group (*n* = 25), 10 and 9 hamsters were immunized with HR121 plus CFA/IFA and CFA/IFA only, respectively, while 6 hamsters were injected with PBS as mock control. Serum samples were collected 7 days after the third immunization. SARS-CoV-2 challenge was carried out 14 days after the third immunization. In LTP group (*n* = 14), 8 and 6 hamsters were injected with HR121 plus CFA/IFA and CFA/IFA only, respectively. Serum samples were collected 83 days after the third immunization. SARS-CoV-2 challenge was carried out 90 days after the third immunization.

For Omicron BA.2 challenge studies, 48 hamsters were divided into 4 groups (12/group). Hamsters in Groups 1 and 2 were injected with HR121 plus CFA/IFA and CFA/IFA only, respectively, while those in Groups 3 and 4 were immunized with HR121/aluminum adjuvant and aluminum adjuvant only, respectively. Serum samples were collected 7 days after the third immunization. Omicron BA.2 challenge was carried out 14 days after the third immunization.

For rhesus macaque (3 males and 5 females, 9–13 years old) vaccination, 4 macaques were vaccinated with 50 μg HR121/IFA at 30-day intervals. Equal numbers of macaques injected with adjuvant were used as controls. Serum samples were collected 7 days after each immunization. SARS-CoV-2 challenge was carried out 7 days after the third immunization.

### Animal passive vaccination

To evaluate the in vivo neutralizing activity of nAbs from HR121-immunized rabbits, sera from the 4 HR121-immunized rabbits were pooled. The IgGs purified from them (~5 mg IgGs obtained from 1 mL sera) were transferred intraperitoneally to 6 hACE2 mice (8 weeks old) or 9 hamsters (8 weeks old) at a dose of 5 mg IgGs/20 g body weight, while isotypical IgGs purified from adjuvant-immunized rabbits were transferred to 4 mice or 8 hamsters as mock controls. 24 h after transfer, SARS-CoV-2 challenge was carried out.

### SARS-CoV-2 challenge

To challenge hACE2 mice vaccinated with HR121 (adjuvant and PBS as controls), the mice were anaesthetized with isoflurane (RWD, Shenzhen, China) and inoculated intranasally with 10^7^ TCID_50_ of SARS-CoV-2 in 30 μL. Lungs were collected 5 days post infection (dpi).

To challenge Syrian golden hamsters vaccinated with HR121, the hamsters were anesthetized and inoculated intranasally with 10^4^ TCID_50_ of SARS-CoV-2 or 10^3^ TCID_50_ of Omicron BA.2 in 100 μL. Lungs were collected at 3 dpi.

To challenge rhesus macaques, the macaques were anesthetized with Zoletil-50 (FeiBo, Beijing, China) and inoculated equally by intranasal and intratracheal routes with 3 × 10^7^ TCID_50_ of SARS-CoV-2 in 2 mL. Lungs were collected at 7 dpi.^97^

To challenge the animals passively administered with rabbit sera, hACE2 mice were inoculated with 10^6^ TCID_50_ of SARS-CoV-2 and Syria golden hamsters were inoculated with 10^4^ TCID_50_ of SARS-CoV-2, respectively. Lungs were collected at 3 dpi.

### bAb detection

A sandwich ELISA was used to detect the bAbs of HR1, HR2, HR12, and HR121 in serum samples. Briefly, HR1, HR2, HR12, or HR121 protein (1 μg/mL) in coating buffer (15 mM Na_2_CO_3_, 35 mM NaHCO_3_, pH 9.6) was coated on 96-well polystyrene plate at 4 °C overnight. After removing the coating buffer, the plate was washed 3 times with PBS containing 0.05% Tween-20 (PBST) and blocked with blocking buffer (PBST containing 5% BSA) at 37 °C for 2 h. Then the plate was washed 3 times, and serially-diluted serum samples were added to the plate and incubated for 2 h at 37 °C. After washing again, OPD substrate was added to each well. The reaction was stopped with 2 M H_2_SO_4_ and the optical density (OD) values of the wells were read on an ELISA reader at 490 nm/630 nm. The endpoint titers of the serum samples were determined as the reciprocal of the last dilution exhibiting an OD value ≥ 2.1-fold that of the average background values.

### HR2/HR121 binding inhibition

Competitive ELISA was used to detect antibodies blocking the binding of HR2 and HR121.^56^ Briefly, HR121 protein (1 μg/mL) was coated onto the ELISA plate at 4 °C overnight. After the coating buffer was removed, the plate was washed with PBST and incubated with blocking buffer at 37 °C for 2 h. Then, the plate was washed 3 times with PBST. Meanwhile, serum or IgG samples from rabbits or humans were serially diluted in PBST and preincubated with HR2-labeled HRP (KPL, Gaithersburg, MD, USA) in 100 ng/mL for 20 min at 20 °C. Then the mixtures were added to each well of the plate and incubated for 1 h at 37 °C. After washing the plate and adding the OPD substrate, the OD values of the plates were determined at 490 nm/630 nm. The percentage inhibition of HR121/HR2 binding was calculated using the following formula: % inhibition = [1 – (E – N)/(P – N)] × 100, where E represents the OD value in the presence of a serum or IgG sample, P represents the OD value in the absence of the serum or IgG sample, and N corresponds to the OD value in the absence of the sample and HR2-labeled HRP. The inhibition data were plotted and log-transformed in GraphPad Prism 8.0.1 (GraphPad Software, Inc., San Diego, CA, USA). The NT_50_s in the serum and IC_50_s in the IgG samples were calculated using the “nonlinear regression (curve fit) — log(inhibitor) vs response -- variable slope (four parameters)” model.

### VSV pseudotyped SARS-CoV-2 env variant production

To construct VSV-based SARS-CoV-2 pseudoviruses, pCMV3 plasmid containing the full sequence of the codon-optimized spike gene was purchased (Sino Biological, Beijing, China). The spike gene with a C-terminal 18-amino acid truncation of the SARS-CoV-2 Wuhan-Hu-1 strain was constructed by PCR and inserted into the eukaryotic expression plasmid pcDNA3.1(+) between the *Bam*H1 and *Eco*R1 sites. A Kozak sequence (GCCACC) was introduced in front of the spike gene to generate the recombinant plasmid pcDNA3.1-SARS-CoV-2-SΔ18.^98^ Based on pcDNA3.1-SARS-CoV-2-SΔ18 sequence, a panel of plasmids containing the SARS-CoV-2 spike-related mutants of S477N, E484K, A222V, N439K, K417N, D614G, and D839Y were introduced using a Fast mutagenesis system kit (Transgen Biotech, Beijing, China). Another panel of plasmids containing the spike genes of 17 important SARS-CoV-2 variants, including B.1.617, B.1.617.1, B.1.617.2.V2, B.1.429, B.1.525, B.1.526, B.1.1.7, B.1.351, B.1.1.28, B.1.617.2, C.37, B.1.621, B.1.1.529 (Omicron BA.1), Omicron BA.2, Omicron BA.3, and Omicron BA.4/5, were synthesized in codon optimization with the corresponding gene fragments. The primers for the point mutants are listed in Supplementary information, Table S2, and the multiple mutations introduced in the spikes of 13 important SARS-CoV-2 variants are listed in Supplementary information, Table S3.

SARS-CoV-2 pseudovirus was prepared using a VSV pseudotyped SARS-CoV-2 S packaging system as described previously.^99^ Briefly, 1 × 10^6^ 293T cells were seeded on a T75 cell flask in DMEM low-sugar medium (Gibco) at 37 °C overnight. After reaching 80% confluence, the cells were transfected with 10 μg pcDNA3.1-SARS-CoV-2-variant-SΔ18 using jetPRIME transfection reagent (Polyplus-transfection, Illkirch, France). After 24 h, the cells were infected with G*ΔG-VSV-Rluc virus at an M.O.I. = 1. Six hours after infection, the cells were washed three times with PBS containing 1% FBS. Thirty-six hours after infection, the supernatant was collected, centrifuged at 1000× *g* for 10 min, aliquoted, and stored at −80 °C. The TCID_50_ of the SARS-CoV-2 pseudotyped virus was determined in 293T-ACE2 cells, as previously described.^99^

### Pseudovirus-based neutralization assays

To measure the neutralizing activity of sera or IgGs against various SARS-CoV-2 variant pseudotyped viruses, 1 × 10^4^ 293T-ACE2 cells (100 μL) were seeded in 96-well plates in DMEM low-sugar medium (Gibco). The next day, the sera from HR121-immunized animals were three-fold serially diluted in another 96-well plate in a volume of 60 μL. Then, 60 μL SARS-CoV-2 pseudovirus (M.O.I. = 0.1) was added to the diluted sera and incubated for 1 h at 37 °C. Thereafter, 100 μL of the mixture was incubated with 293T-ACE2 cells for 24 h at 37 °C, and the supernatant was removed from the cells after incubation. Renilla luciferase activity was determined using a Renilla luciferase assay kit (Promega, Madison, WI, USA). The NT_50s_ of the sera or IC_50_s of the IgGs were calculated in GraphPad Prism 8.0.1 software as mentioned above.

### SARS-CoV-2-based neutralization assays

8 × 10^5^ HPAEpiC cells (200 μL) were seeded into each well of a 48-well plate and incubated at 37 °C overnight. The next day, sera or IgGs from HR121-immunized animals were two-fold serially diluted in another 48-well plate at a volume of 100 μL. Then, 100 μL SARS-CoV-2 (M.O.I. = 1) were added into the diluted sera or IgG and incubated for 1 h at 37 °C. Thereafter, the medium was removed and replaced with the virus-serum or virus-IgG mixture. After incubating at 37 °C for another 1 h, the mixture was removed and washed 3 times with PBS. Subsequently, fresh medium (200 μL) containing the same diluted sera or IgGs was added. The cells were cultured at 37 °C for 48 h, and then the supernatants were collected for viral RNA extraction by kit (Roche Diagnostics, Mannheim, Germany), followed by analysis of viral load (viral genome RNA). The NT_50_ and IC_50_ values were calculated using GraphPad Prism 8.0.1 as mentioned above.

### Viral load measurement

RNA was extracted from lung tissues using Trizol (Life Technologies, Carlsbad, CA, USA). The RNA concentration was measured using a NanoDrop 2000 (Thermo Fisher Scientific, USA). Viral gRNA and sgRNA were measured by real-time qPCR using a one-step qRT-PCR kit (QRZ-101, Toyobo, Osaka, Japan) on a ViiA7 Real-Time PCR System (Life Technologies). The primers for gRNA detection were derived from the nucleocapsid (*N*) gene of SARS-CoV-2, as previously described,^88^ including forward, 5′-GGGGAACTTCTCCTGCTAGAAT-3′; reverse, 5′-CAGACATTTTGCTCTCAAGCTG-3′; and probe, 5′-FAM-TTGCTGCTGCTTGACAGATT-TAMRA-3′. The primers for sgRNA detection were derived from the envelope (*E*) gene of SARS-CoV-2, as previously described,^7^ including forward, 5′-CGATCTCTTGTAGATCTGTTCTC-3′; reverse, 5′-ATATTGCAGCAGTACGCACACA-3′; and probe, 5′-FAM-CGAAGCGCAGTAAGGATGGCTAGTGT-TAMRA-3′.

### Real-time qPCR

Real-time qPCR was performed to evaluate the mRNA expression of inflammation-related cytokines in the lung tissues of hACE2 mice after SARS-CoV-2 challenge. Primers (for genes including *IFNG*, *IL-2*, *IL-4*, *IL-6*, *IL-10*, *IP-10*, *MX2*, and *TNFA*) were designed according to mouse mRNA sequences (Supplementary information, Table S4). cDNA was generated using a PrimeScript RT Reagent Kit with gDNA Eraser (Takara, Beijing, China). Real-time qPCR was performed using a ViiA7 Real-Time PCR System and SYBR Premix Ex Taq II (Takara). Expression levels of the genes of interest were analyzed using the comparative cycle threshold (Ct) method, where Ct is the cycle threshold number normalized to that of *ACTB* mRNA. The fold change was calculated using the 2^−ΔΔCt^ method by dividing the normalized quantity of post-infection samples by that of healthy hACE2 mouse samples.

### Histopathology and immunohistochemistry

Lung tissues of SARS-CoV-2-infected animals were collected and fixed in 4% paraformaldehyde for 3 days, followed by embedding in paraffin and cutting into 3 μm sections. Some sections were stained with H&E for evaluation of lung injury, and other sections were stained with the SARS-CoV-2 nucleocapsid protein (Sino Biological) to evaluate SARS-CoV-2 replication levels in the lung tissues.

### ELISpot assays

SARS-CoV-2 HR1-specific cytotoxic T lymphocytes (CTLs) were evaluated using a murine IFN gamma set (Diaclone Research, Besancon, France) according to the manufacturer’s instructions. Briefly, a MultiScreenHTS IP filter plate (Millipore) was pre-coated with anti-murine IFNγ. Then, 1 × 10^6^ splenocytes isolated from BALB/c mice were co-cultured with a pool of 15-amino-acid overlapped peptides covering the full HR1 sequence in 1 μg/mL (Supplementary information, Table S5, synthesized by Generay Biotech) for 24 h. After incubation, the wells were washed and colored, and images were collected using an ImmunoSpot S6 universal analyzer (Cellular Technology Limited, Cleveland, OH, USA). Using an automated program, the spots were counted with parameters such as size, intensity and gradient.

To evaluate SARS-CoV-2 HR121-specific humoral responses, a MultiScreenHTS IP filter plate was pre-coated with HR121 (1 μg/mL), and washed 3 times. Then, 1 × 10^6^ splenocytes isolated from BALB/c mice were added into each well and cultured for 24 h. Using the same method as that for IFNγ, the plate was imaged.

### Statistics

All statistical analyses were performed using GraphPad Prism 8.0.1. *P* values were labeled in the figures. The titers of the bAbs or nAbs were presented as geometric mean ± geometric SD, and the copies of viral gRNAs or sgRNAs were presented as median ± interquartile range. Two-tailed Mann-Whitney test or Wilcoxon test was used to compare the difference between HR121 and control groups.

## Supplementary information

Supplementary information, Fig. S1

Supplementary information, Fig. S2

Supplementary information, Fig. S3

Supplementary information, Fig. S4

Supplementary information, Fig. S5

Supplementary information, Fig. S6

Supplementary information, Fig. S7

Supplementary information, Fig. S8

Supplementary information, Table S1

Supplementary information, Table S2

Supplementary information, Table S3

Supplementary information, Table S4

Supplementary information, Table S5

Supplementary information, Video S1

Supplementary Video S1 legend

## Data Availability

The data of HR121 crystal structure have been deposited at https://deposit-pdbj.wwpdb.org/deposition with PDB ID: 7WXZ. This paper does not report original code. Any additional information required to reanalyze the data reported in this paper is available from the lead contact upon request.
