# Supplementary material for: A variant-proof SARS-CoV-2 vaccine targeting HR1 domain in S2 subunit of spike protein
Source: Cell Res. 2022 Nov 10;32(12):1068–85. doi: 10.1038/s41422-022-00746-3 (PMC9648449; doi:10.1038/s41422-022-00746-3)
Supplement: Supplementary file 1 — Supplementary information, Fig. S1 [file 41422_2022_746_MOESM1_ESM.pdf]

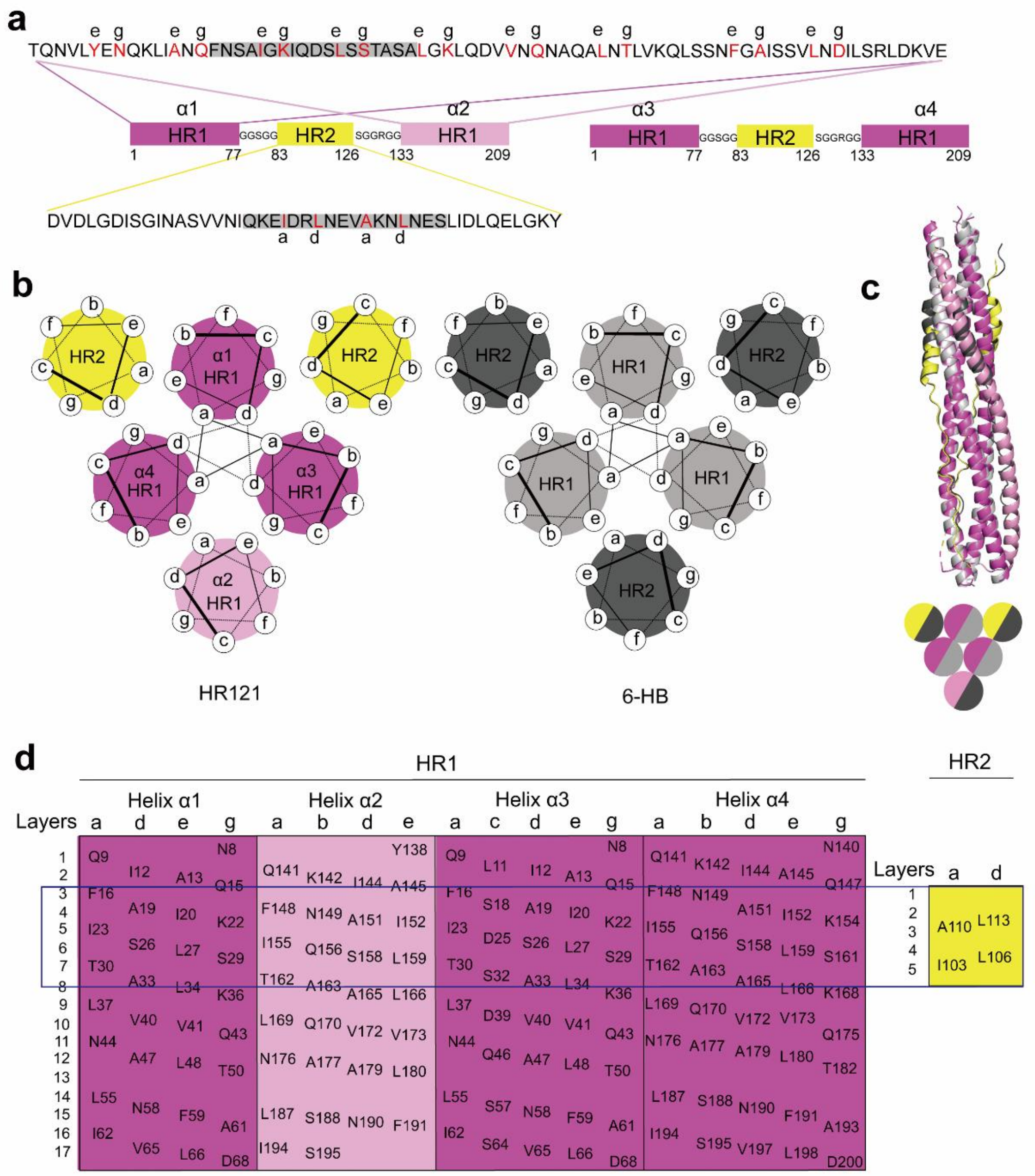
a
e g e g e g e g e g e g e g e g e g
TQNVLYENQKLIANQFNSAIGKIQDSLSSTASALGKLQDVVNQNAQALNTLVKQLSSNFGAISSVLNDILSRLDKVE
α1
α2
α3
α4
HR1
GGSGG
HR2
SGGRGG
HR1
1 77 83 126 133 209
DVDLGDISGINASVVNIQKEIDRLNEVAKNLNESLIDLQELGKY
a d a d
b
HR2
α1 HR1
α2 HR1
α3 HR1
α4 HR1
HR121
HR2
HR1
6-HB
c
d
HR1
HR2
Helix α1
Helix α2
Helix α3
Helix α4
Layers
a d e g a b d e a c d e g a b d e g
1 2 3 4 5 6 7 8 9 10 11 12 13 14 15 16 17
Q9 I12 A13 N8 Q15 F16 A19 I20 K22 I23 S26 L27 S29 T30 A33 L34 K36 L37 V40 V41 Q43 N44 A47 L48 T50 L55 N58 F59 A61 I62 V65 L66 D68
Y138 Q141 K142 I144 A145 F148 N149 A151 I152 I155 Q156 S158 L159 T162 A163 A165 L166 L169 Q170 V172 V173 N176 A177 A179 L180 L187 S188 N190 F191 I194 S195
Q9 L11 I12 A13 N8 Q15 F16 S18 A19 I20 K22 I23 D25 S26 L27 S29 T30 S32 A33 L34 K36 L37 D39 V40 V41 Q43 N44 Q46 A47 L48 T50 L55 S57 N58 F59 A61 I62 S64 V65 L66 D68
Q141 K142 I144 A145 N140 Q147 F148 N149 A151 I152 K154 I155 Q156 S158 L159 S161 T162 A163 A165 L166 K168 L169 Q170 V172 V173 Q175 N176 A177 A179 L180 T182 L187 S188 N190 F191 A193 I194 S195 V197 L198 D200
Layers a d
1 2 3 4 5
A110 L113 I103 L106

Supplementary information, Fig. S1: Interhelical packing interactions in HR121. **a** Schematic representation of HR121. The corresponding sequences of HR1, HR2 and the linker are shown in diagram. The “e” and “g” positions in HR1 and “a” and “d” residues in HR2 are colored in red. The residues involved in interaction between HR1 and HR2 are marked with a grey background. **b** Left: formation of HR121 between HR1 and HR2. The inner HR1 trimer (α1, α3, and α4) is formed through the interaction of residues located at the “a” and “d” positions, and two HR2s depend on the residues located at the “a” and “d” positions to interact with the residues in “e” and “g” positions in the inner HR1 trimer. Another HR1 (α2) depend on the residues at “e” positions to bind with the residues at “g” positions in HR1 (α3), and the residues at “a” positions to bind with the residues at “e” positions in HR1 (α4). Right: formation of the post-fusion structure of SARS-CoV-2 6-HB (PDB code: 6LXT). The HR1 trimer is formed through the interaction of residues located at “a” and “d” positions, and the HR2 depend on the residue located at the “a” and “d” position to interact with the residues in “e” and “g” positions in HR1. **c** A cartoon representation showing superimposition of the inner HR1 trimer (α1, α3, and α4) of HR121 with the central HR1 trimer of SARS-CoV2 6-HB (PDB code: 6LXT). The figures below are diagrams of the superimposed helices in top view. **d** List of interacting residues between HR121 helical bundles. The helical fusion core regions are marked with dark blue box.
