# Supplementary material for: A variant-proof SARS-CoV-2 vaccine targeting HR1 domain in S2 subunit of spike protein
Source: Cell Res. 2022 Nov 10;32(12):1068–85. doi: 10.1038/s41422-022-00746-3 (PMC9648449; doi:10.1038/s41422-022-00746-3)
Supplement: Supplementary file 2 — Supplementary information, Fig. S2 [file 41422_2022_746_MOESM2_ESM.pdf]

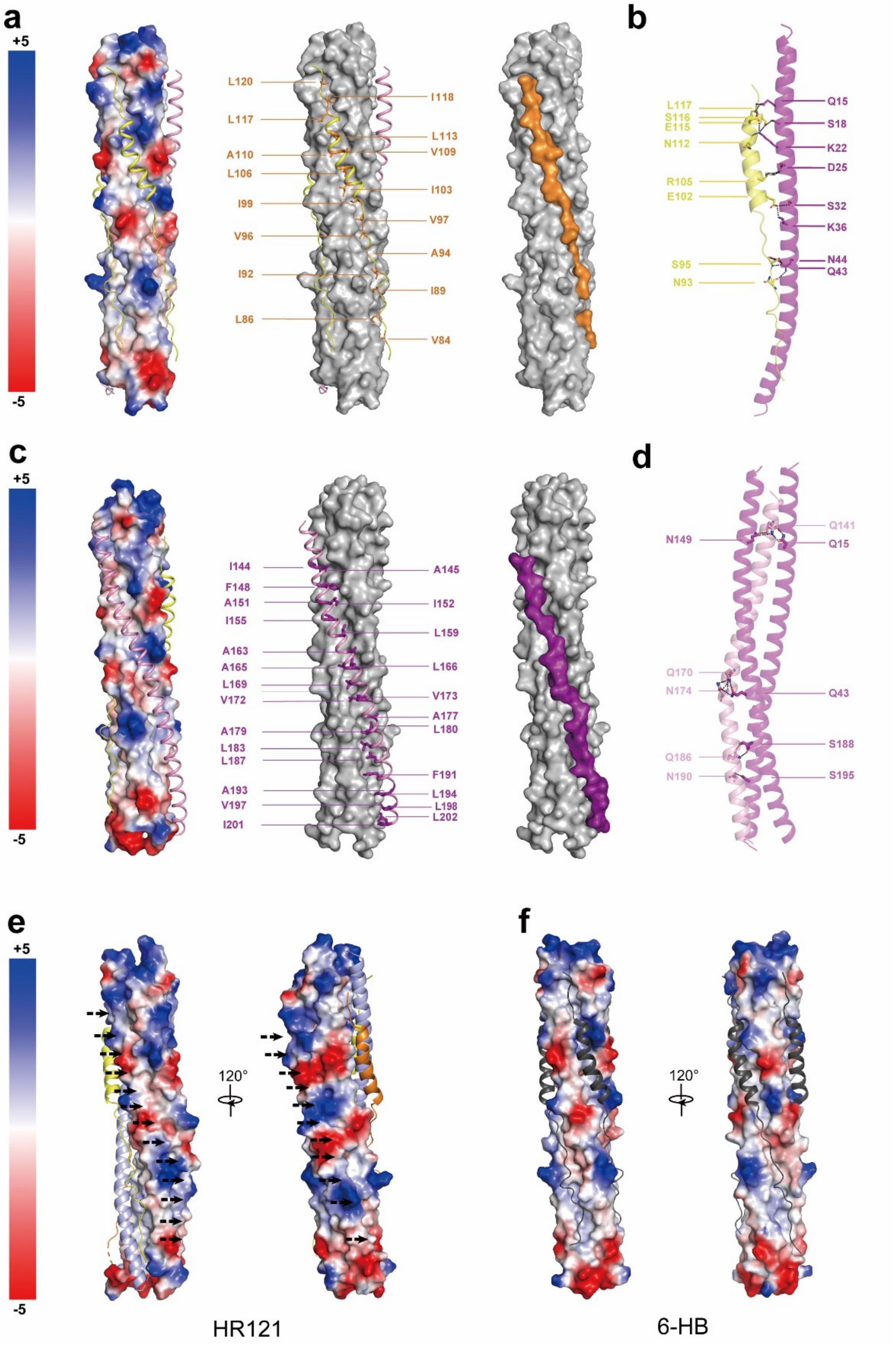
a
+5
-5
L120
L117
A110
L106
I99
V96
I92
L86
I118
L113
V109
I103
V97
A94
I89
V84
b
L117
S116
E115
N112
R105
E102
S95
N93
Q15
S18
K22
D25
S32
K36
N44
Q43
c
+5
-5
I144
F148
A151
I155
A163
A165
L169
V172
A179
L183
L187
A193
V197
I201
A145
I152
L159
L166
V173
A177
L180
F191
L194
L198
L202
d
N149
Q141
Q15
Q170
N174
Q43
Q186
N190
S188
S195
e
+5
-5
120°
HR121
f
120°
6-HB

Supplementary information, Fig. S2: Interaction between the outer helices of HR2s and HR1 (α2) with inner HR1 trimer (α1, α3, and α4).

a Left: Electrostatic potential surface of the inner HR1 trimer (α1, α3 and α4). Hydrophobic surfaces are colored in whitish gray, basic in blue, and acidic in red, the same in other panels. Each of two adjacent HR1 helices forms a hydrophobic groove, in which an HR2 (shown in cartoon representation and colored in orange) is embedded. Middle: Hydrophobic interactions between HR2 and the inner HR1 trimer. Key hydrophobic residues in an HR2 binding to the inner HR1 trimer are indicated by orange sticks and labeled. Right: HR2 hydrophobic residues involved in hydrophobic interactions with the inner HR1 trimer. **b** Hydrophilic interaction between an HR2 and an HR1 in the inner HR1 trimer. Residues that mediate the interaction of the HR1 and HR2 are indicated by sticks and marked aside. hydrogen bonds are labeled in dark grey lines. **c** Left: The HR1 (α2, colored in purple) shown in cartoon representation embedded in hydrophobic groove of the inner HR1 trimer. Middle: Hydrophobic interactions between the HR1 (α2) and the inner HR1 trimer. Key hydrophobic residues in the HR1 (α2) are indicated by purple sticks and labeled. Right: theHR1 (α2) hydrophobic residues involved in hydrophobic interactions with the inner HR1 trimer. **d** Hydrophilic interaction between the HR1 (α2) and two HR1s in the inner HR1 trimer. Residues that mediate the interaction of the HR1 and HR2 are indicated by sticks and marked aside. hydrogen bonds are labeled in dark grey lines. **e** Electrostatic surface on the two exposed surface grooves on the outer HR1 trimer of HR121. These two grooves are covered highly charged amino acid residues. The outer HR1 trimer of HR121 are surrounded by one HR1(α1, colored in light greys) and two HR2s (colored in orange and dark orange, respectively). The right image is the result of rotating the left image 120° toward the viewer. Black arrows point to the possible HR2 or antibody binding grooves. **f** Electrostatic surface grooves on the HR1 trimer of SARS-CoV-2 6-HB. The two corresponding HR2s (which are lacked in HR121) in cartoon mode (colored in black) are embedded in the hydrophobic grooves in the HR1 trimer. The right image is the result of rotating the left image 120°toward the viewer.
