# Supplementary material for: A variant-proof SARS-CoV-2 vaccine targeting HR1 domain in S2 subunit of spike protein
Source: Cell Res. 2022 Nov 10;32(12):1068–85. doi: 10.1038/s41422-022-00746-3 (PMC9648449; doi:10.1038/s41422-022-00746-3)
Supplement: Supplementary file 3 — Supplementary information, Fig. S3 [file 41422_2022_746_MOESM3_ESM.pdf]

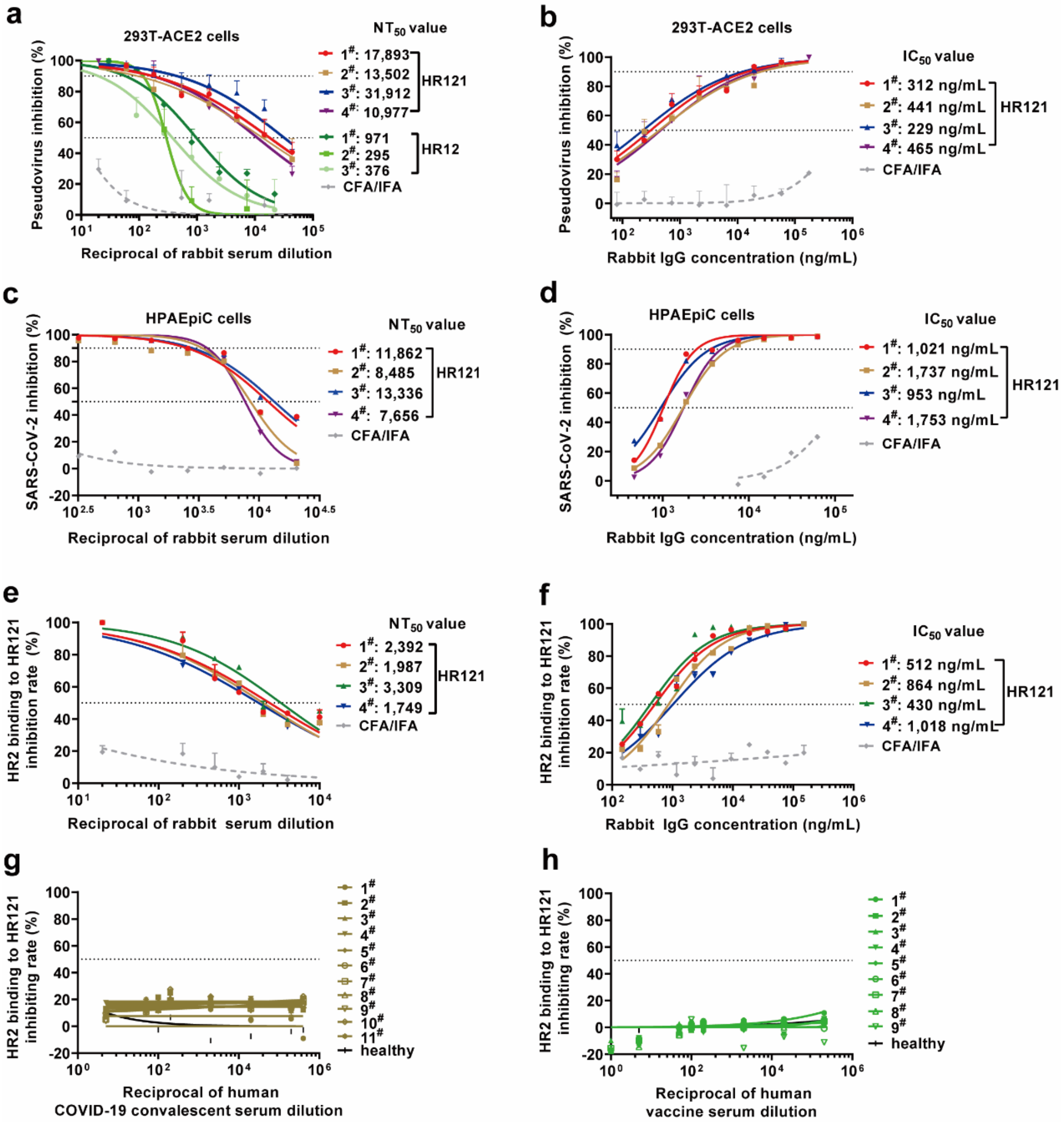

Supplementary information, Fig. S3: Rabbit anti-HR121 sera and IgG could potently inhibit both SARS-CoV-2 replication and HR2 binding to HR121 in dose-dependent manners.

a, **b** SARS-CoV-2 pseudovirus entering 293T-ACE2 cells was potently inhibited in a dose-dependent manner by rabbit anti-HR121 sera (**a**) and IgG (**b**). Here, rabbit anti-HR12 sera were set as controls in the pseudovirus assay. **c**, **d** SARS-CoV-2 replication in HPAEpiC cells was also blocked in a dose-dependent manner by rabbit anti-HR121 sera (**c**) and IgG (**d**). **e**, **f** HR2 binding to HR121 was potently inhibited by rabbit anti-HR121 sera (**e**) and IgG (**f**). **g**, **h** HR2 binding to HR121 was not disturbed by sera from human COVID-19 convalescents (**g**) and vaccinated individuals (**h**).
