# Supplementary material for: A variant-proof SARS-CoV-2 vaccine targeting HR1 domain in S2 subunit of spike protein
Source: Cell Res. 2022 Nov 10;32(12):1068–85. doi: 10.1038/s41422-022-00746-3 (PMC9648449; doi:10.1038/s41422-022-00746-3)
Supplement: Supplementary file 4 — Supplementary information, Fig. S4 [file 41422_2022_746_MOESM4_ESM.pdf]

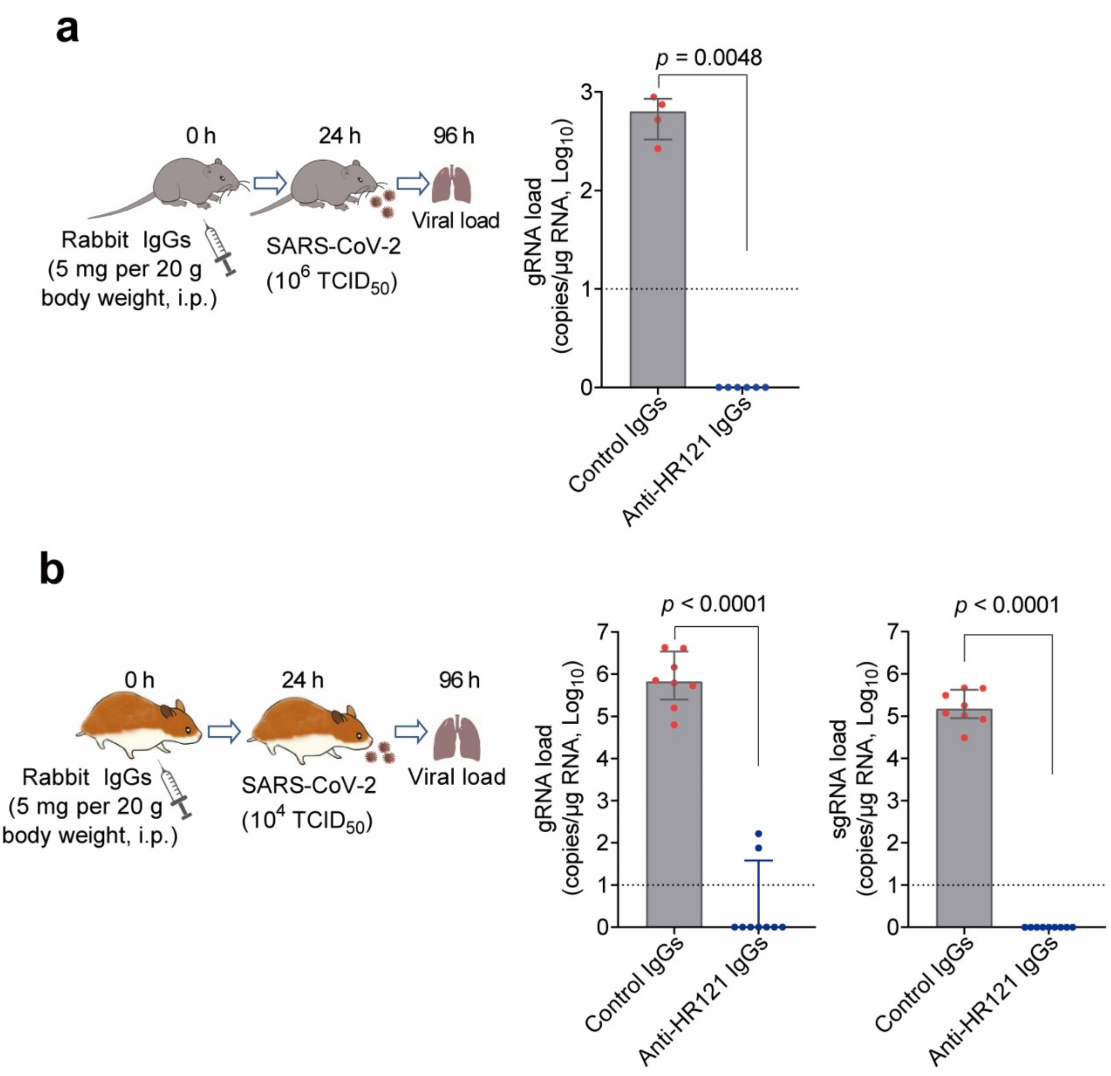

Supplementary information, Fig. S4: Rabbit anti-HR121 IgGs could protect hACE2 mice (a) and Syrian golden hamsters (b) from SARS-CoV-2 infection. SARS-CoV-2 gRNAs and sgRNAs were quantified by quantitative PCR with reverse transcription (RT–qPCR); each point represents a mouse or hamster; data are presented as median ± interquartile range; p values are determined by two-tailed Mann-Whitney test.
