# Supplementary material for: A variant-proof SARS-CoV-2 vaccine targeting HR1 domain in S2 subunit of spike protein
Source: Cell Res. 2022 Nov 10;32(12):1068–85. doi: 10.1038/s41422-022-00746-3 (PMC9648449; doi:10.1038/s41422-022-00746-3)
Supplement: Supplementary file 5 — Supplementary information, Fig. S5 [file 41422_2022_746_MOESM5_ESM.pdf]

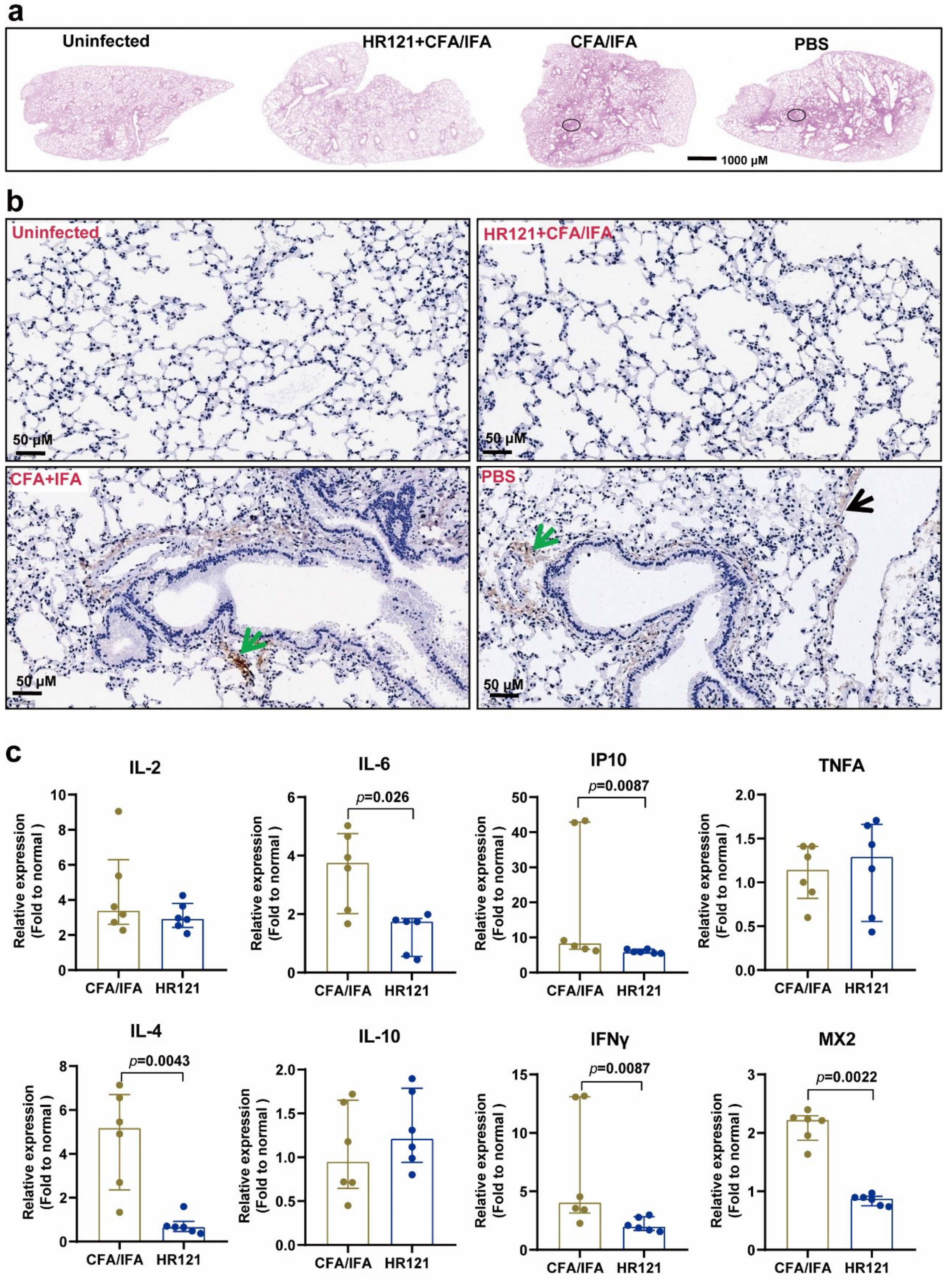
a
Uninfected
HR121+CFA/IFA
CFA/IFA
PBS
1000 μM
b
Uninfected
HR121+CFA/IFA
CFA+IFA
PBS
50 μM
c
IL-2
IL-6
IP10
TNFA
IL-4
IL-10
IFNγ
MX2
Relative expression (Fold to normal)
CFA/IFA
HR121
p=0.026
p=0.0087
p=0.0043
p=0.0087
p=0.0022

Supplementary information, Fig. S5: Representative histopathological and cytokine gene changes in lung tissues of SARS-CoV-2-infected hACE-2 mice.

a Scanning images of whole lung tissue sections by H&E staining. A representative image from uninfected hACE-2 mouse (n = 1), and SARS-CoV-2-infected hACE-2 mice vaccinated with HR121 plus CFA/IFA (n = 3), vaccinated with CFA/IFA (n = 3) and PBS (n = 3). **b** Nucleocapsid protein of SARS-CoV-2 immunostaining. In the mice immunized with HR121, no nucleocapsid proteins were detected after SARS-CoV-2 challenge, just as in uninfected mice. Meanwhile, in the CFA/IFA and PBS controls, nucleocapsid proteins were stained and, thus, detected around bronchial epithelial cells (black arrow) and alveolar epithelia (green arrow). A representative image from each group (n = 3) is shown. **c** Some antiviral and proinflammatory cytokine gene expression changes. Of 6/8 mice vaccinated with HR121, and 6/8 mice injected with CFA/IFA were selected for evaluation. Non-infected controls (n = 3). Data are presented as median ± interquartile range (two tailed Mann-Whitney test).
