# Supplementary material for: A variant-proof SARS-CoV-2 vaccine targeting HR1 domain in S2 subunit of spike protein
Source: Cell Res. 2022 Nov 10;32(12):1068–85. doi: 10.1038/s41422-022-00746-3 (PMC9648449; doi:10.1038/s41422-022-00746-3)
Supplement: Supplementary file 6 — Supplementary information, Fig. S6 [file 41422_2022_746_MOESM6_ESM.pdf]

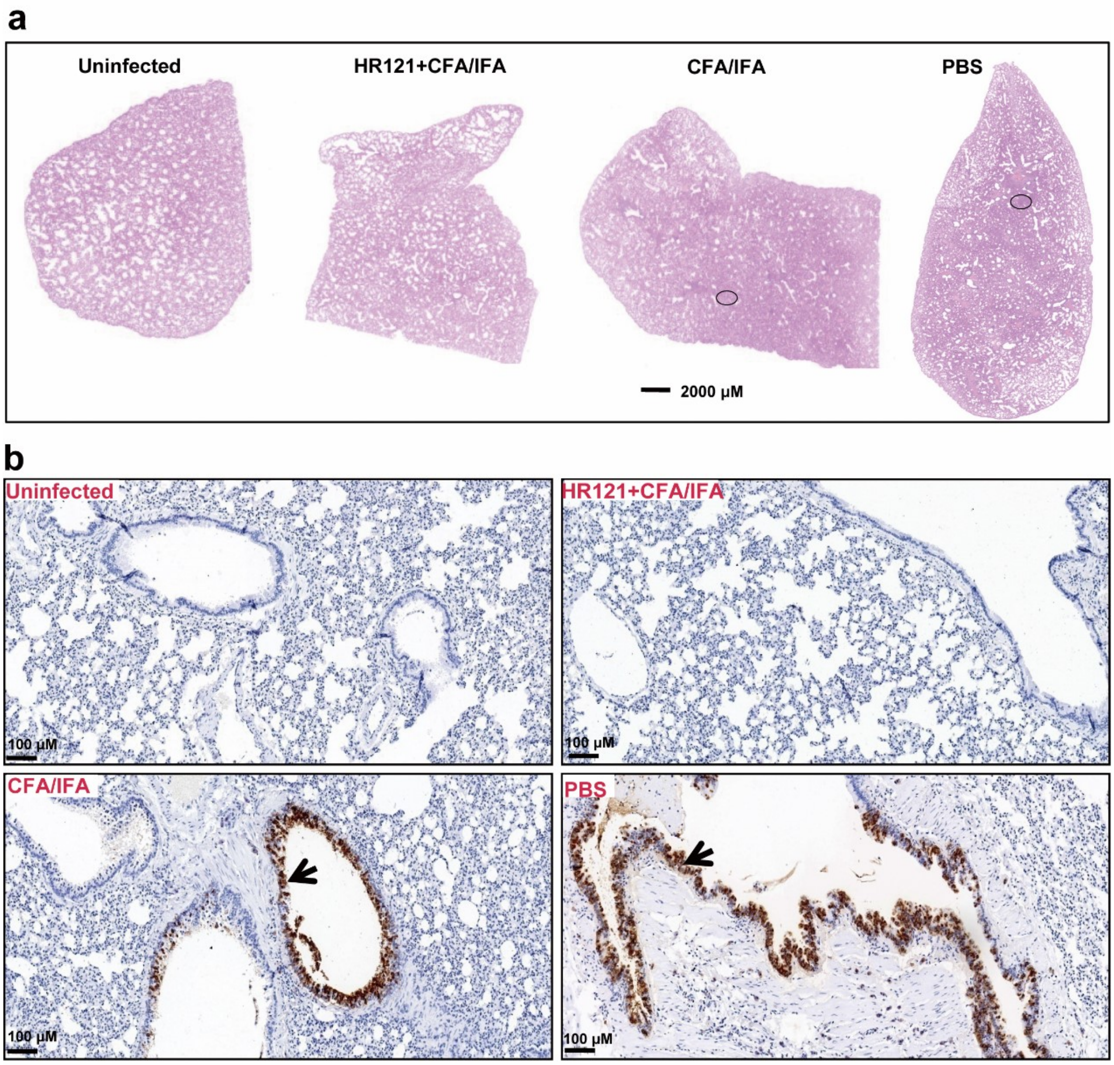

Supplementary information, Fig. S6: Representative histopathological changes in lung tissues of SARS-CoV-2-infected Syrian golden hamsters.

a Scanning images of whole lung tissue sections by H&E staining. A representative image from uninfected Syrian golden hamsters (n = 4), and SARS-CoV-2-infected Syrian golden hamsters vaccinated with HR121 plus CFA/IFA (n = 6), vaccinated with CFA/IFA (n = 6) and PBS (n = 6). **b** Nucleocapsid protein of SARS-CoV-2 immunostaining. In the hamsters immunized with HR121, no nucleocapsid proteins were detected after SARS-CoV-2 challenge, just as in uninfected hamsters. Meanwhile, in the groups of CFA/IFA and PBS controls, more stained nucleocapsid proteins were detected around bronchial epithelial cells (black arrow). A representative photograph of each group (n = 3) is presented.
