# Supplementary material for: A variant-proof SARS-CoV-2 vaccine targeting HR1 domain in S2 subunit of spike protein
Source: Cell Res. 2022 Nov 10;32(12):1068–85. doi: 10.1038/s41422-022-00746-3 (PMC9648449; doi:10.1038/s41422-022-00746-3)
Supplement: Supplementary file 7 — Supplementary information, Fig. S7 [file 41422_2022_746_MOESM7_ESM.pdf]

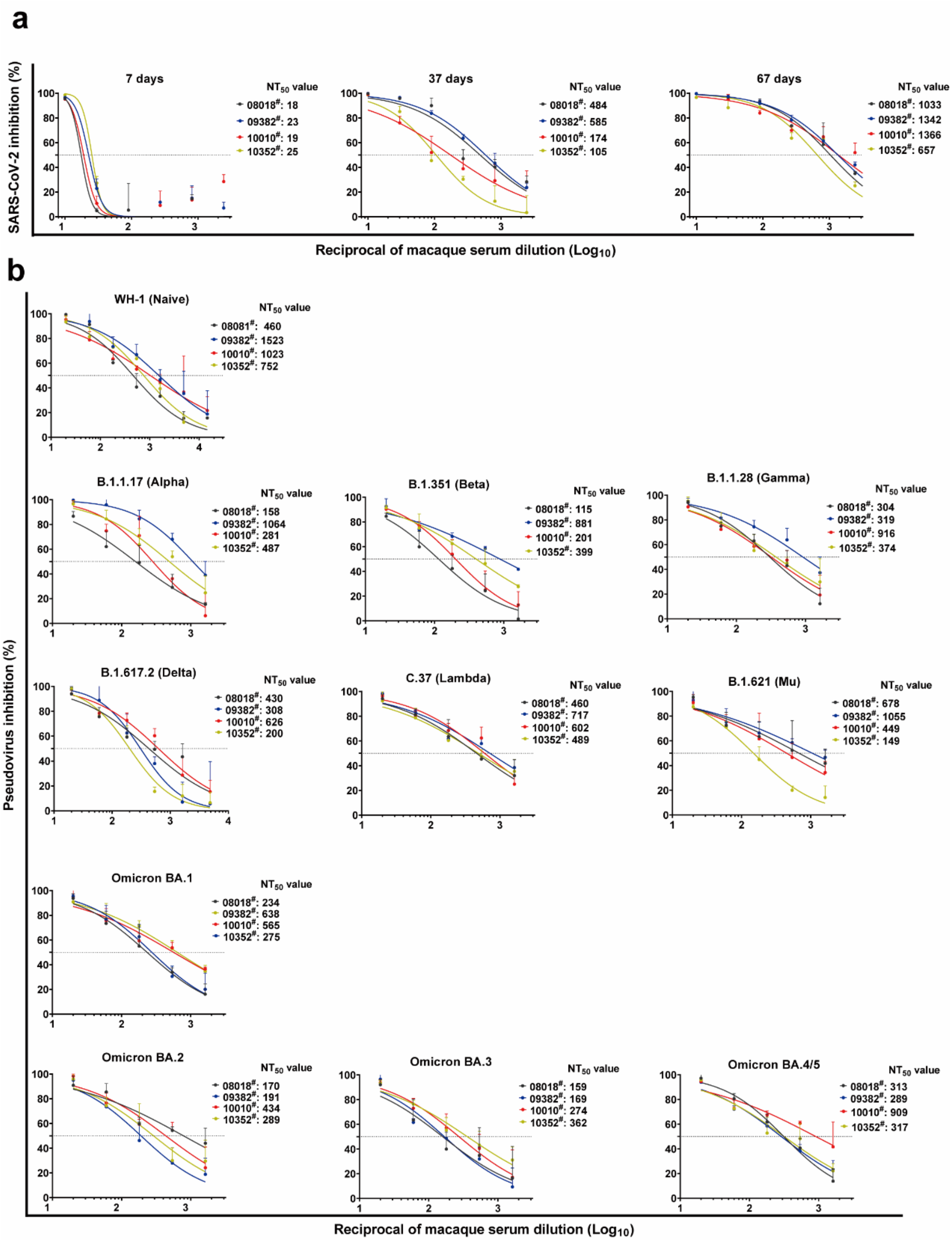

Supplementary information, Fig. S7: Rhesus macaques anti-HR121 sera demonstrated broadly antiviral activities against SARS-CoV-2 and its main ancestral and current variants.

a SARS-CoV-2 replication in HPAEpiC cells were inhibited in a dose-dependent manner by macaque anti-HR121 sera post 7 days of each immunization. **b** VSV-based pseudoviruses enveloped with different SARS-CoV-2 spikes entering 293T-ACE2 cells were inhibited in a dose-dependent manner by macaque anti-HR121 sera.
