# Supplementary material for: A variant-proof SARS-CoV-2 vaccine targeting HR1 domain in S2 subunit of spike protein
Source: Cell Res. 2022 Nov 10;32(12):1068–85. doi: 10.1038/s41422-022-00746-3 (PMC9648449; doi:10.1038/s41422-022-00746-3)
Supplement: Supplementary file 8 — Supplementary information, Fig. S8 [file 41422_2022_746_MOESM8_ESM.pdf]

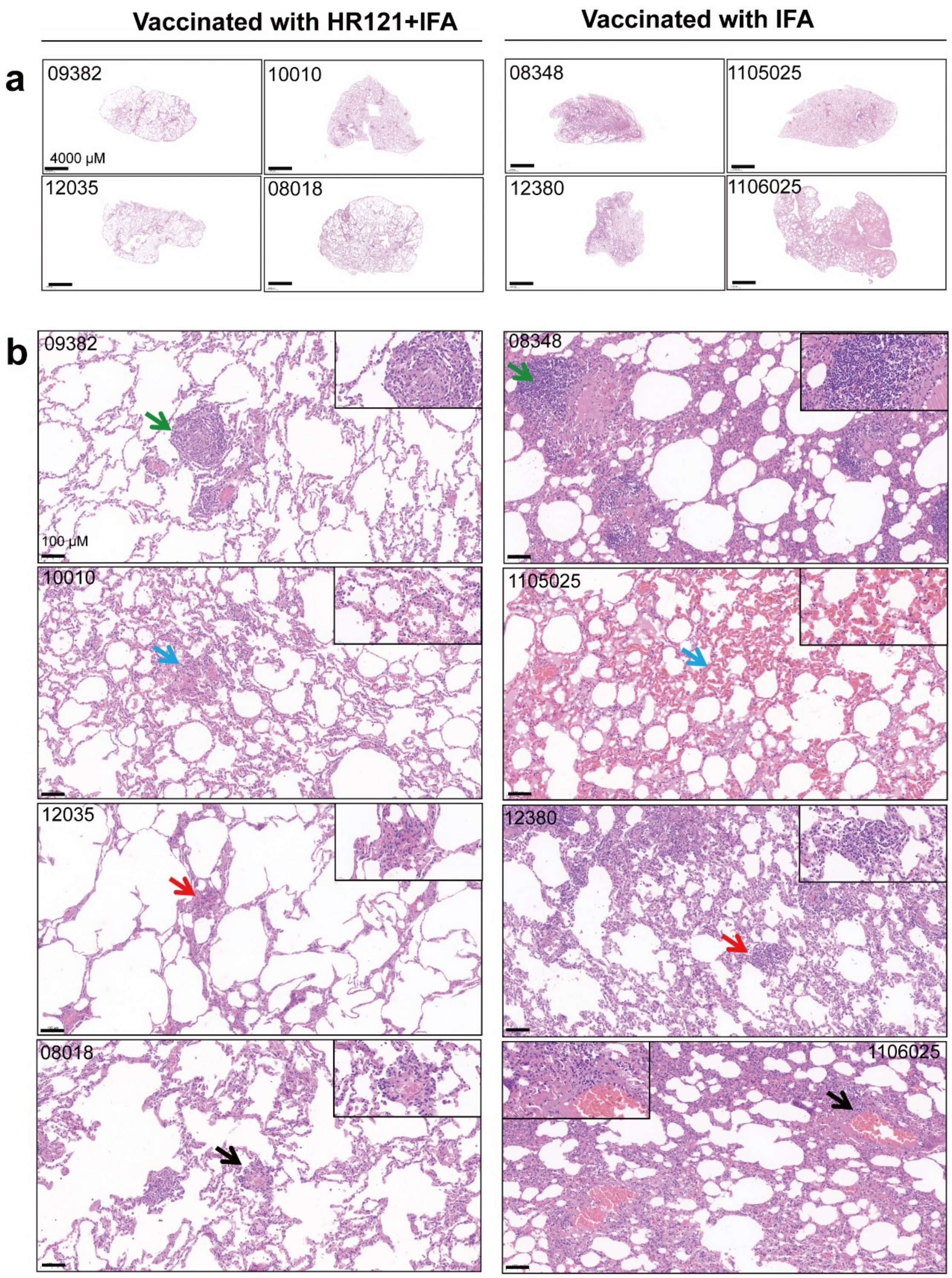
Vaccinated with HR121+IFA
Vaccinated with IFA
a
09382
10010
08348
1105025
4000 μM
12035
08018
12380
1106025
b
09382
08348
100 μM
10010
1105025
12035
12380
08018
1106025

Supplementary information, Fig. S8: Representative histopathological changes in lung tissues of SARS-CoV-2-infected rhesus macaques.

A representative lung tissue section by H&E staining from each rhesus macaque infected with SARS-CoV-2 is presented. Four macaques (ID: 09382, 10010, 12035, 08018) were vaccinated with HR121 plus IFA, and four macaques (ID: 08348, 1105025, 12380, 1106025) were vaccinated with IFA. At least three tissue sections (from upper, middle, and lower lung lobes) were assessed per macaque. **a** A representative scanning image of whole lung tissue section by H&E staining from each rhesus macaque. **b** Representative histopathology in macaques vaccinated with HR121 plus IFA or IFA only. In each layer, the typical histopathological lesions, such as lymphoid proliferation (green arrow), intra-alveolar hemorrhage (blue arrow), alveolar macrophage infiltrates (red arrow), and perivascular macrophage infiltrates (black arrow) are marked.
