# Supplementary material for: A variant-proof SARS-CoV-2 vaccine targeting HR1 domain in S2 subunit of spike protein
Source: Cell Res. 2022 Nov 10;32(12):1068–85. doi: 10.1038/s41422-022-00746-3 (PMC9648449; doi:10.1038/s41422-022-00746-3)
Supplement: Supplementary file 9 — Supplementary information, Table S1 [file 41422_2022_746_MOESM9_ESM.pdf]

Supplementary information, Table S1: Data collection and refinement statistics

**Data collection**	**SARS-CoV-2 HR121**
**Space group**	**P 1 2 1**
**Cell dimensions**	
a, b, c (Å)	65.16, 51.73, 119.16
α, β, γ (°)	90.00, 95.33, 90.00
Wavelength (Å)	0.9791
Resolution (Å)	32.46 - 2.41 (32.55 - 2.41)
R_{merge}	0.02889 (0.9947)
$CC_{1/2}$	1 (0.434)
Mean I/σ(I)	10.62 (0.84)
Completeness (%)	99.46 (99.58)
Redundancy	2.0 (2.0)
**Refinement**	
Resolution (Å)	32.46-2.41
Total reflections	61592 (6140)
R_{work}/R_{free}	0.2489/0.2913 (0.4572/0.5382)
**No. of atoms**	
Protein	757
Water & ligands	0
**Average B-factor ($Å^2$)**	
Overall	91.01
Water & ligands	0
**R.m.s. deviations**	
Bond lengths (Å)	0.013
Bond angles (°)	1.53
Ramachandran outliers (%)	10.64
**Ramachandran plot (%)**	
Favored region	99.18
Allowed region	0.82
Disallowed region	0.00

*Highest resolution shell is shown in parentheses.
