# Supplementary material for: A variant-proof SARS-CoV-2 vaccine targeting HR1 domain in S2 subunit of spike protein
Source: Cell Res. 2022 Nov 10;32(12):1068–85. doi: 10.1038/s41422-022-00746-3 (PMC9648449; doi:10.1038/s41422-022-00746-3)
Supplement: Supplementary file 10 — Supplementary information, Table S2 [file 41422_2022_746_MOESM10_ESM.pdf]

Supplementary information, Table S2: Primers to generate point mutants in the recombinant plasmid pcDNA3.1-SARS-CoV-2-SΔ18.

point mutants	Forward primer (5’-3’)	Reverse Primer (5’-3’)
D614G	TGGCTGTGCTCTACCAGGGAGTGAACTGTA	TCCCTGGTAGAGCACAGCCACCTGGTTGCT
S477N	AGATTTACCAGGCTGGCAACACACCATGTA	GTTGCCAGCCTGGTAAATCTCTGTGCTGAT
A222V	TGCCACAGGGCTTCTCTGTCTTGGAACCAC	GACAGAGAAGCCCTGTGGCAGGTCCCTCAC
E484K	CACCATGTAATGGAGTGAAGGGCTTCAACT	CTTCACTCCATTACATGGTGTGCTGCCAGC
K417N	CCCCTGGACAAACAGGCAACATTGCTGACT	GTTGCCTGTTTGTCCAGGGGCAATCTGTCT
D839Y	TCATCAAGCAATATGGATACTGTCTGGGAG	GTATCCATATTGCTTGATGAAGCCAGCATC
N439K	TGATTGCCTGGAACAGCAACAACCTGGACA	GAACGACAAGGTCCGTTAGTGTGTCGGACA
