# Supplementary material for: A variant-proof SARS-CoV-2 vaccine targeting HR1 domain in S2 subunit of spike protein
Source: Cell Res. 2022 Nov 10;32(12):1068–85. doi: 10.1038/s41422-022-00746-3 (PMC9648449; doi:10.1038/s41422-022-00746-3)
Supplement: Supplementary file 11 — Supplementary information, Table S3 [file 41422_2022_746_MOESM11_ESM.pdf]

Supplementary information, Table S3: SARS-CoV-2-S circulating variants constructed in this study.

SARS-CoV-2-S circulating variants	Mutation sites
B.1.617.1	G142D, E154K, L452R, E484Q, D614G, P618R, Q1071H, and H1101D
B.1.617.2.V2	T19R, G142D, E156G, 157-158 FR deletion (ΔFR157-158), L452R, T478K, D614G, P681R and D950N
B.1.429	S13I, W152C, and L452R
B.1.525	Q52R, A67V, ΔHV69-70, ΔY145, E484K, D614G, Q677H, and F888L
B.1.526	L5F, T95I, D253G, E484K, D614G, and A701V
B.1.617	E154K, L452R, E484Q, and P681R
B.1.1.7 (Alpha)	Δ69–70 HV, Δ144 Y, N501Y, A570D, D614G, P681H, T761I, S982A, and D1118H
B.1.351 (Beta)	L18F, D80A, D215G, ΔLAL242-244, K417N, E484K, N501Y, D614G, and A701V
B.1.1.28 (Gamma)	L18F, T20N, P26S, D138Y, R190S, K417T, E484K, N501Y, D614G, and H655Y.
B.1.617.2 (Delta)	T19R, G142D, L452R, T478K, D614G, P681R and D950N
C.37 (Lambda)	G75V，T76I，ΔR246，ΔS247，ΔY248，ΔL249，ΔT250，ΔP251，ΔG252，D253N，L452Q，F490S，D614G，and T859N
B.1.621 (Mu)	T95I, Y146insN, R346K, E484K, N501Y, D614G, P681H, and D950N
B.1.1.529 (Omicron BA.1)	A67V, ΔHV69-70, T95I, ΔGVY142-144, Y145D, ΔN211, L212I, ins214EPE, G339D, S371L, S373P, S375F, K417N, N440K, G446S, S477N, T478K, E484A, Q493R, G496S, Q498R, N501Y, Y505H, T547K, D614G, H655Y, N679K, P681H, N764K, D796Y, N856K, Q954H, N969K, and L981F
Omicron BA.2	T19I; ΔL24, ΔP25, ΔP26, A27S, G142D, V213G, G339D, S371F, S373P, S375F, T376A, D405N, R408S, K417N, N440K, S477N, T478K, E484A, Q493R, Q498R, N501Y, Y505H, D614G, H655Y, N679K, P681H, N764K, D796Y, Q954H, N969K
Omicron BA.3	A67V, Δ69-70, T95I, G142D, ΔV143, ΔY144, ΔY145, ΔN211, L212I, G339D, S371F, S373P, S375F, D405N, K417N, N440K, G446S, S477N, T478K, E484A, Q493R, Q498R, N501Y, Y505H, D614G, H655Y, N679K, P681H, N764K, D796Y, Q954H, N969K
Omicron BA.4/5	T19I, L24S, Δ25-27, Δ69-70, G142D, V213G, G339D, S371F, S373P, S375F, T376A, D405N, R408S, K417N, N440K, L452R, S477N, T478K, E484A, F486V, Q498R, N501Y, Y505H, D614G, H655Y, N679K, P681H, N764K, D796Y, Q954H, N969K
