# Supplementary material for: A variant-proof SARS-CoV-2 vaccine targeting HR1 domain in S2 subunit of spike protein
Source: Cell Res. 2022 Nov 10;32(12):1068–85. doi: 10.1038/s41422-022-00746-3 (PMC9648449; doi:10.1038/s41422-022-00746-3)
Supplement: Supplementary file 12 — Supplementary information, Table S4 [file 41422_2022_746_MOESM12_ESM.pdf]

Supplementary information, Table S4: Primers for amplifying inflammation related cytokines in the lung tissues of hACE2 mice.

Genes	GenBank ID	Forward primer (5'-3')	Reverse primer (5'-3')
mouse *ACTB*	NM_007393.5	GATATCGCTGCGCTGGTCG	CATTCCCACCATCACACCCT
mouse *IFNG*	NM_008337.4	CAGCAAGGCGAAAAAGGATGC	CTTCCTGAGGCTGGATTCCG
mouse *IL-2*	NM_008366.3	GAAACTCCCCAGGATGCTCA	CGCAGAGGTCCAAGTTCATCT
mouse *IL-4*	NM_021283.2	CCAAACGTCCTCACAGCAAC	AGGCATCGAAAAGCCCGAA
mouse *IL-6*	NM_001314054.1	AACCAAGAGATAAGCTGGAGTCAC	AACGCACTAGGTTTGCCGAG
mouse *IL-10*	NM_010548.2	CCTGGGTGAGAAGCTGAAGAC	CTTGTAGACACCTTGGTCTTGG
mouse *IP-10*	NM_021274.2	TGCAGGATGATGGTCAAGCC	CCACTTGAGCGAGGACTCAG
mouse *MX2*	NM_013606.1	GTCGCCTATTCACCAGGCTC	TCGTCCACGGTACTGCTTTT
mouse *TNFA*	NM_013693.3	ATGGCCTCCCTCTCATCAGT	TTTGCTACGACGTGGGCTAC
