# Supplementary material for: A variant-proof SARS-CoV-2 vaccine targeting HR1 domain in S2 subunit of spike protein
Source: Cell Res. 2022 Nov 10;32(12):1068–85. doi: 10.1038/s41422-022-00746-3 (PMC9648449; doi:10.1038/s41422-022-00746-3)
Supplement: Supplementary file 13 — Supplementary information, Table S5 [file 41422_2022_746_MOESM13_ESM.pdf]

Supplementary information, Table S5: The peptide sequences of the HR1 domain.

Serial No.	Peptides	Length (amino acid)
1	TQNVLYENQKLIANQ	15
2	LIANQFNSAIGKIQD	15
3	GKIQDSLSSTASALG	15
4	ASALGKLQDVVNQNA	15
5	VNQNAQALNTLVKQL	15
6	LVKQLSSNFGAISSV	15
7	AISSVLNDILSRLDK	15
8	SRLDKVE	7
