# Supplementary material for: A variant-proof SARS-CoV-2 vaccine targeting HR1 domain in S2 subunit of spike protein
Source: Cell Res. 2022 Nov 10;32(12):1068–85. doi: 10.1038/s41422-022-00746-3 (PMC9648449; doi:10.1038/s41422-022-00746-3)
Supplement: Supplementary file 15 — Supplementary Video S1 legend [file 41422_2022_746_MOESM15_ESM.pdf]

Supplementary information, Video S1 HR121 monomer consists of two parallel HR1s packed with one antiparallel HR2. The two HR121 monomers aggregate in parallel together in an asymmetric manner; thus, HR121 dimer is formed with four parallel HR1s surrounded by two antiparallel HR2s.
